# Changes in Serum *N*-Glycome for Risk Drinkers: A Comparison with Standard Markers for Alcohol Abuse in Men and Women

**DOI:** 10.3390/biom12020241

**Published:** 2022-02-01

**Authors:** Róisín O’Flaherty, Ádám Simon, Manuela Alonso-Sampedro, Sonia Sánchez-Batán, Carmen Fernández-Merino, Francisco Gude, Radka Saldova, Arturo González-Quintela

**Affiliations:** 1 GlycoScience Group, National Institute for Bioprocessing Research and Training, Fosters Avenue, Mount Merrion, Blackrock, A94 X099 Co. Dublin, Ireland; roisin.oflaherty@mu.ie (R.O.); simonadam9085@gmail.com (Á.S.); radka.fahey@nibrt.ie (R.S.); 2 Department of Chemistry, Maynooth University, Maynooth, W23 F2K8 Co. Kildare, Ireland; 3 Grupo de Metodología de la Investigación, Instituto de Investigación Sanitaria (IDIS), Complejo Hospitalario Universitario, Santiago de Compostela, 15706 Santiago de Compostela, Spain; manuela.alonso.sampedro@sergas.es (M.A.-S.); sonia.sanchez.batan@sergas.es (S.S.-B.); francisco.gude.sampedro@sergas.es (F.G.); 4 Primary Care Center, A Estrada, 36680 Pontevedra, Spain; carmen.fernandez.merino@sergas.es; 5 Department of Clinical Epidemiology, Complejo Hospitalario Universitario, Santiago de Compostela, 15706 Santiago de Compostela, Spain; 6 UCD School of Medicine, College of Health and Agricultural Science, University College Dublin, D04 V1W8 Dublin 4, Ireland; 7 Department of Internal Medicine, Complejo Hospitalario Universitario, University of Santiago de Compostela, 15706 Santiago de Compostela, Spain

**Keywords:** alcohol, risk drinking, *N*-glycome, *N*-glycan, glycomics, public health, biomarker, diagnosis, serum

## Abstract

**Background and aim**: Glycomic alterations serve as biomarker tools for different diseases. The present study aims to evaluate the diagnostic capability of serum *N*-glycosylation to identify alcohol risk drinking in comparison with standard markers. **Methods**: We included 1516 adult individuals (age range 18–91 years; 55.3% women), randomly selected from a general population. A total of 143 (21.0%) men and 50 (5.9%) women were classified as risk drinkers after quantification of daily alcohol consumption and the Alcohol Use Disorders Identification Test (AUDIT). Hydrophilic interaction ultra-performance liquid chromatography (HILIC-UPLC) was used for the quantification of 46 serum *N*-glycan peaks. Serum gamma-glutamyltransferase (GGT), carbohydrate-deficient transferrin (CDT), and red blood cell mean corpuscular volume (MCV) were measured by standard clinical laboratory methods. **Results**: Variations in serum *N*-glycome associated risk drinking were more prominent in men compared to women. A unique combination of *N*-glycan peaks selected by the *selbal* algorithm shows good discrimination between risk-drinkers and non-risk drinkers for men and women. Receiver operating characteristics (ROC) curves show accuracy for the diagnosis of risk drinking, which is comparable to that of the golden standards, GGT, MCV and CDT markers for men and women. Additionally, the inclusion of *N*-glycan peaks improves the diagnostic accuracy of the standard markers, although it remains relatively low, due to low sensitivity. For men, the area under the ROC curve using *N*-glycome data is 0.75, 0.76, and 0.77 when combined with GGT, MCV, and CDT, respectively. In women, the areas were 0.76, 0.73, and 0.73, respectively. **Conclusion**: Risk drinking is associated with significant variations in the serum *N*-glycome, which highlights its potential diagnostic utility.

## 1. Introduction

According to the 2019 WHO report, 3 million deaths worldwide are related to harmful alcohol use. Furthermore, above two hundred health conditions from cancer to liver diseases, motor vehicle accidents, and criminal offences related to harmful alcohol use [1]. From a clinical point of view, it is important to detect alcohol use disorders and risk drinking. Acute alcohol consumption can be assessed by measuring ethanol concentration in the blood, but chronic alcohol consumption has to be evaluated by the use of biomarkers with a much longer half-life than ethanol. Standard markers include serum gamma-glutamyl transferase (GGT), mean corpuscular volume of erythrocytes (MCV), and carbohydrate-deficient transferrin (CDT) [2,3,4,5,6]. Both GGT and MCV can be routinely determined in the clinical laboratory but have limited sensitivity and specificity since they can be influenced by non-alcohol-related disorders [2,3,4]. Moreover, the use of MCV is limited due to the long lifespan of the erythrocytes, which diminishes its potential as a relapse marker [4].

Glycosylation is the most common co- and post-translational modification of proteins and peptides in mammals [7,8]. It has an essential role in the proper folding, stability, solubility and effector function of proteins. Glycans are involved in virtually all biological processes and in the pathophysiology of every major disease in humans [8]. Therefore, glycosylation changes can be used as biomarkers, often with high accuracy for a wide array of diseases [7,8], particularly cancer [9,10,11,12,13]. Glycosylation is also affected by age, sex [14,15] and lifestyle, including diet [16] or smoking [15]. Along this line, the percentage of CDT to total serum transferrin is an approved biomarker for the detection of chronic alcohol abuse [3,17]. The most abundant form of transferrin contains disialylated biantennary glycan structures [18]. The loss of sialylation can be detected by analytical methods [19] and is indicative of chronic alcohol abuse [3]. At the cellular level, this loss of sialylation can be explained by the inhibitory effect of acetaldehyde (a metabolite of ethanol) on some of the sialyltransferase, galactosyltransferase and *N*-acetylglucosaminyltransferase activity in the Golgi apparatus in liver cells [20]. Acetaldehyde and ethanol cause Golgi remodelling and inhibit the activity of enzymes, which are important to create certain glycan structures [21,22,23]. 

Experience with CDT shows that glycomic analyses could be a promising tool for the detection of alcohol risk drinking. Despite the diagnostic potentials of glycomics in general [8,24], its accuracy to identify heavy alcohol intake has not yet been fully addressed. The serum *N*-glycome, (i.e., the set of all glycans that are *N*-linked to asparagine in serum proteins) is well studied due to the availability of a specific enzyme (PNGase F) for the *N*-glycan release and the availability of associated analytical methods for their proper separation and identification [25,26]. In this study, we aimed to address the potential of serum *N*-glycome as a biomarker of heavy alcohol consumption. The performance of the most common biomarkers (GGT, CDT, and MCV) was also investigated to compare their accuracy with that of the *N*-glycans.

## 2. Methods

### 2.1. Study Design

This cross-sectional study was developed in the municipality of A-Estrada (Northwestern Spain, location 42°41′21″ N, 8°29′14″ W). An outline of the study (AEGIS, A-Estrada Glycation and Inflammation Study) is available at www.clinicaltrials.gov (Accessed on 19 January 2022), code NCT01796184 and detailed elsewhere [27]. The municipality had an adult population (age >18 years) of 18474 when the study started in 2012. An age-stratified random sample of the population aged 18 years and older was drawn from Spain’s National Health System Registry, which covers more than 95% of the population and contains the name, date of birth and address of every person entitled to primary care. From an initial sample of 3500 individuals, 2230 could be assessed for eligibility and displayed no exclusion criteria; of these, 1516 individuals agreed to participate (overall participation rate, 68%). Participation was lower in men than in women (65% vs. 71%). There were no significant differences in terms of age or residence (rural vs. urban) between participants and non-participants. From November 2012 to March 2015, all subjects were successively contacted and asked to attend the Primary Care Centre for evaluation, which included an interviewer-administered structured questionnaire and fasting venous blood sampling. Median age of participants was 52 years (range, 18–91 years) and 838 (55.3%) were women. Basic characteristics of men and women are summarised in Table 1. 

### 2.2. Ethical Issues

Written informed consent was obtained from all participants. The general survey and the specific glycomic studies were approved by the Galician Regional Ethics Committee (codes 2010-315 and 2016-464, respectively) and conformed to the current Helsinki Declaration. 

### 2.3. Assessment of Smoking

Consumers of at least one cigarette per day were deemed to be smokers. Individuals who had quit smoking during the preceding year were still considered smokers. Ex-smokers and those that never smoked were grouped together for this study.

### 2.4. Definition of Alcohol Risk Drinking

The habitual alcohol consumption was evaluated in standard drinking units [28], by summing the number of glasses of wine (~10 g), bottles of beer (~10 g), and units of spirits (~20 g) regularly consumed per week. All participants also underwent an AUDIT (Alcohol Use Disorders Identification Test) questionnaire, which was validated in Spain [29,30,31]. Risk alcohol drinking for women was considered when regular alcohol consumption was greater than 20 g/day or the AUDIT score was greater than 5 points. Risk alcohol drinking for men was considered when regular alcohol consumption was greater than 40 g/day or the AUDIT score was greater than 7 points [29,30,31,32,33,34]. With these criteria, the prevalence of risk drinking was higher in men (143 of 678, 21.0%) than in women (50 of 838, 5.9%). Alcohol intake was higher in men than in women, both among risk drinkers and among non-risk drinkers (Table 1).

### 2.5. Serum Collection

Blood was drawn after overnight fasting using BD Vacutainer^®^SST Serum Separation Tubes (BD, Plymouth, UK). After collection, tubes were inverted five times, allowed 30 min clotting time, and centrifuged at room temperature for 15 min at 1300× *g* in a fixed-angle centrifuge. The serum was removed, aliquoted and stored at −80 °C for further use. 

### 2.6. Determination of Serum GGT, MCV and CDT

Gamma-glutamyl transferase (GGT) was determined in fresh serum samples from fasting participants on a fully automatic Analyser (ADVIA 2400, Siemens Healthcare Diagnostics, Barcelona, Spain) by a standard method recommended by the International Federation of Clinical Chemistry (IFCC). Reference range for GGT in adults is 8–73 IU/L for men and 4–38 IU/L for women.

Mean corpuscular volume of red blood cells (MCV) was determined in fresh blood samples on an ADVIA 2120 automated hematology Analyser (Siemens Healthcare Diagnostics, Barcelona, Spain). Reference range for MCV in adults is 80–100 fL.

Carbohydrate-deficient transferrin (CDT) was determined after thawing frozen serum samples using the commercial CAPILLARYS CDTTM kit on a MINICAP CDTTM device (Sebia, Lisses, France) a multicapillary electrophoresis system, carefully following manufacturer’s instructions. Transferrin isoforms are separated in silica capillaries by their electrophoretic mobility and electro-osmotic flow at high voltage in an alkaline buffer. Transferrin isoforms are directly detected during migration by UV absorbance. The serum transferrin isoforms are separated into 5 main fractions according to their level of sialylation (asialotransferrin (non-sialylated), disialotransferrin, trisialotransferrin, tetrasialotransferrin and pentasialotransferrin). The proportion of each fraction is calculated as the area under the curve. The low sialylation transferrin isoforms (that is, asialotransferrin and disialotransferrin; there are usually no detectable monosialylated forms) constitute the CDT, the value of which is calculated automatically by the system. Genetic variants and abnormal profiles were well recognised. In cases of abnormal peak profiles or other interferences in the electropherogram, samples were remeasured following sample cleanup by sample treatment as specified by the manufacturer. The manufacturer recommends the following interpretation of results: normal (CDT ≤ 1.3%), indeterminate (CDT > 1.3% and ≤ 1.6%), and abnormal or indicative of alcohol abuse (CDT > 1.6%).

### 2.7. Serum N-Glycan Analyses

*N*-glycans were released from 5 μL of serum samples using a modified high-throughput automated method [35]. Briefly, the samples were denaturated with dithiothreitol (Sigma Aldrich, Arklow, Ireland), alkylated with iodoacetamide (Sigma Aldrich, Arklow, Ireland), and *N*-glycans were released from the protein backbone enzymatically via PNGase F (NEB, Ipswich, MA, USA, recombinant, code P0709L, 10 μL per well, 5000 U) in 1 M ammonium bicarbonate, pH 8.0 (Sigma Aldrich, Arklow, Ireland). Glycans were then immobilised on solid supported hydrazide beads on 96-well chemically inert filter plate (Millipore Solvinert, hydrophobic polytetrafluoroethylene membrane, 0.45 μm pore size, Millipore Ireland B.V., Carrigtwohill, Ireland), and excess reagents were removed by centrifuge filtration. Glycans were released from the solid supports and labelled with the fluorophore 2-aminobenzamide (2-AB, Sigma Aldrich, Arklow, Ireland). Glycans were cleaned up using HyperSep Diol SPE cartridges (Thermo Fisher Scientific, Waltham, MA, USA). 

For hydrophilic interaction chromatography (HILIC) ultra-performance liquid chromatography (UPLC), fluorescently labelled *N*-glycans were separated on a Waters Acquity H-Class UPLC instrument (Waters, Milford, MA, USA) consisting of a quaternary solvent manager, sample manager-FTN, column manager and an FLR fluorescence detector set with excitation and emission wavelengths of 330 and 420 nm, respectively. The instrument was under the control of Empower 3 software, build 3471 (Waters, Milford, MA, USA). Labelled *N*-glycans were separated on a Waters Ethylene Bridged Hybrid BEH Glycan chromatography column, 150 × 2.1 mm i.d., 1.7 μm BEH particles (Waters, Milford, MA, USA), with 50 mM ammonium formate pH 4.4 (made from formic acid, Sigma Aldrich, Arklow, Ireland), and 25% ammonium hydroxide (Sigma Aldrich, Arklow, Ireland) as solvent A and acetonitrile (MeCN, Sigma Aldrich, Arklow, Ireland) as solvent B. The column was fitted with an ACQUITY in-line 0.2 μm filter. Separation method used linear gradient of 70–30% acetonitrile (*v/v*) at flow rate of 0.56 mL/min in a 30 min analytical run. Samples were maintained at 4 °C before injection. An injection volume of 25 µL sample prepared in 70% *v/v* MeCN was used throughout. Samples were maintained at 5 °C prior to injection and the separation temperature was 40 °C. The system was calibrated using an external standard of hydrolysed and 2-AB labelled glucose oligomers to create a dextran ladder, as described previously [36]. A fifth-order polynomial distribution curve was fitted to the dextran ladder to assign glucose unit (GU) values from retention times (using Empower software from Waters. Milford, MA, USA). The chromatograms were all separated in the same manner into 46 peaks according to Saldova et al. [12], and the amount of glycans in each peak was expressed as % of total integrated area. Glycan structures were annotated using the SNFG nomenclature and the DrawGlycan-SNFG software (University at Buffalo, Buffalo, NY, USA) [37,38] with the assist of GlycoStore.org (accessed on 11 November 2021) [39].

A summary of glycan peaks (GPs) and the corresponding *N*-glycan structures can be found in Supplementary Table S1. Groups of GPs were defined from their common features, as follows [12]:

**Sialylation**: S0 (neutral (asialylated), GP1−15); S1 (monosialylated, GP16−23 + GP30); S2 (disialylated, GP24−29 + GP31); S3 (trisialylated, GP32−40); S4 (tetrasialylated, GP41−46). 

**Galactosylation**: G0 (agalactosylated, GP1−2 + GP4−5 + (GP6/2) + (GP12/2)); G1 (monogalactosylated, GP3 + GP7−10 + (GP12/2) + GP16−18 + (GP21/2)); G2 (digalactosylated, GP13−15 + GP19 + GP20 + (GP21/2) + GP22−28); G3 (trigalactosylated, GP29 + GP31−37); G4 (tetragalactosylated, GP30 + GP38−46). 

**Branching**: A1 (monoantennary, GP1−3 +(GP12/2) + (GP21/2)); A2 (biantennary, GP4−5 + (GP6/2) + GP7−10 + (GP12/2) + GP13−20 + (GP21/2) + GP22−28); A3 (triantennary, GP29 + GP31−37); A4 (tetraantennary, GP30 + GP38−46). 

**Oligomannose**: (GP6/2) + GP11. 

**Fucosylation**: Core-fucose (CF) (GP2 + GP5 + (GP6/2) + GP8−10 + GP14−15 + GP17−18 + GP22−23 + GP27−28 + GP36 + (GP44/2)) and outer-arm fucose (OF) (GP37 + GP40 + (GP41/3) + GP45 + (GP46/3)).

Mass spectrometry-assisted glycan characterisation was performed for two representative samples and a technical replicate-otherwise the major glycans were identified and assigned based on their GU values cross-referenced in Glycobase, now migrated to Glycostore and based on previous assignments in Saldova et al. [12]. Online coupled fluorescence (FLR)-mass spectrometry detection was performed on Phynexus purified samples using a Waters Xevo G2 QTof with Acquity^®^ UPLC (Waters Corporation, Milford, MA, USA) and BEH Glycan column (1.0 × 150 mm, 1.7 μm particle size). For MS acquisition data the instrument was operated in positive-sensitivity mode with a capillary voltage of 2.3 kV. The ion source block and nitrogen desolvation gas temperatures were set at 120 °C and 400 °C, respectively. The desolvation gas was set to a flow rate of 600 L/h. The cone voltage was maintained at 20 V. Full-scan data for glycans were acquired over m/z range from 450 to 2500. Data collection and processing were controlled by MassLynx 4.1 software (Waters Corporation, Milford, MA, USA). The fluorescence detector settings were as follows: λexcitation: 330 nm, λemission: 420 nm; data rate was 1pts/second and a PMT gain = 10. Sample injection volume was 10 μL (75% MeCN). The flow rate was 0.150 mL/min and column temperature was maintained at 60 °C; solvent A was 50 mM ammonium formate (pH 4.4) and solvent B was MeCN. A 40 min linear gradient was used and was as follows: 28% A for 1 min, 28–43% A for 30 min, 43–70% A for 1 min, 70% A for 3 min, 70–28% solvent A for 1 min and 28% A for 4 min. To avoid contamination of system, flow was sent to waste for the first 1.2 min and after 32 min. A summary of *N*-glycan structures identified by mass spectrometry can be found in Supplementary Table S2. 

### 2.8. Statistical Analyses

All analyses were stratified by sex as the criteria for risk drinking were different in men and women, the criteria for normality of some markers were also different, and there were significant differences in the proportions of the different GPs between men and women (data not shown). We employed the Mann–Whitney test to compare the numerical data between groups. We employed the Chi-squared test to compare proportions and the Jonckheere–Terpstra test for trend analysis of numerical variables among ordinal categories. For univariate statistical analyses involving GPs, both uncorrected and sequential Bonferroni-adjusted *p*-values are shown.

To identify the best combination of glycan peaks (GPs) which is predictive for risk drinking, we used the R package *selbal*, a method for analysis of compositional data, i.e., data consisting of quantitative descriptions of the parts of some whole (as the sum of the 46 serum GPs, which is 100% or 1), conveying relative information. The method performs multiple regressions a number of times, each time adding a new GP to the model. Unlike linear regression, the raw variables in *selbal* are not included in a linear equation in real space but are added as part of what is called a “balance” in the compositional data analysis literature, i.e., as part of a particular type of log-contrast. Balances between different sample types are detected by identifying the smallest number of differentially variables that is predictive of sample condition. This method was proposed by Rivera-Pinto et al. [40] for the identification of signatures from compositional data that are predictive of a phenotype of interest [41]. The *selbal* algorithm is available on GitHub at https://github.com/malucalle/selbal/archive/master.zip (accessed on 19 January 2022).

The area under the curve (AUC) from the receiver operating characteristics (ROC) analysis was used to assess the predictive performance of GPs and commercial markers of risk drinking status (CDT, GGT and MCV), after adjusting for age and smoking status. The ROC curves and the AUC, with 95% confidence intervals (95% CI) were calculated using the “pROC” package [42].

## 3. Results

The results of the commercial markers of alcohol consumption in risk drinkers and non-risk drinkers are shown in Table 2. All markers (GGT, MCV and CDT) were significantly higher in risk drinkers compared to non-risk drinkers in men and women. However, their diagnostic accuracy was limited. The sensitivity of these markers (i.e., the proportion of risk drinkers with the altered marker) in men ranged from 7.1% for MCV, 18.2% for GGT to 25.9% for CDT. In women, however, the highest sensitivity was for GGT (32.0%), followed by MCV (10.0%) and the lowest sensitivity was for CDT (8.0%). The specificity of the markers (i.e., the proportion of non-risk drinkers with a negative test) was higher. In men, the best specificity was for MCV (98.7%), followed by CDT (94.4%) and GGT (81.8%). In women, the best specificity was also for MCV (99.9%), and CDT (99.5%), and it was lower for GGT (91.2%) (Table 2). Accordingly, the positive likelihood ratio for the detection of risk drinking in men was 2.03 (95% CI, 1.30–3.15) for GGT, 4.61 (95% CI, 2.96–7.20) for CDT, and 5.34 (95% CI 2.07–14.0) for MCV. In women, the positive likelihood ratio of GGT was higher (3.65, 95% CI 2.30–5.80). The positive likelihood ratios of CDT and MCV were also higher, but the small number of cases with a positive test made the confidence intervals very broad (16.0 (95% CI 4.06–61.0), and 79 (95% CI 9.38–662), respectively).

**Figure 1:**
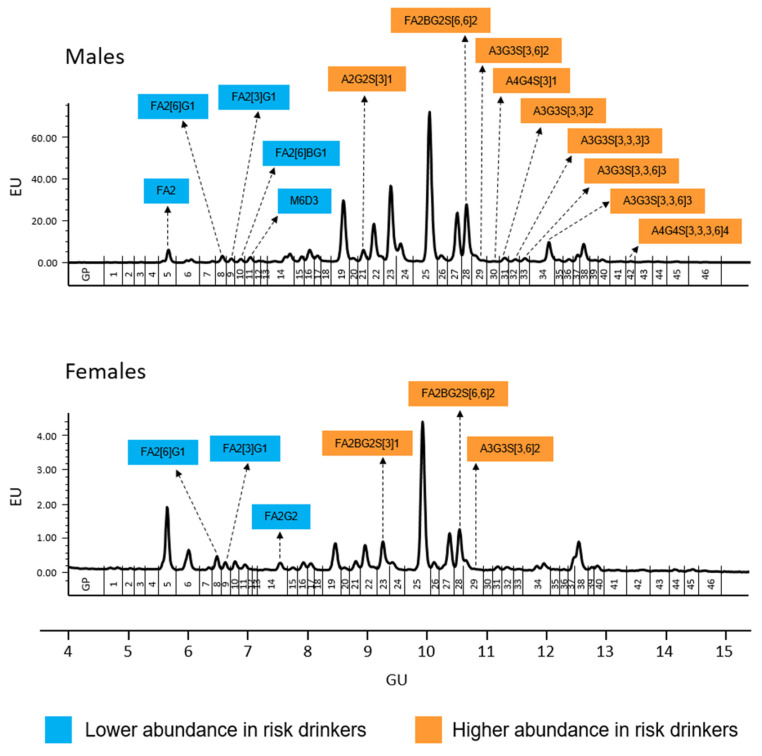
Serum *N*-glycome representative chromatograms in men (upper panel) and women (lower panel). Glycan peaks (GPs) are numbered from GP1–GP46 and assigned as in Saldova et al. [12]. The integration areas, together with a major structure presented in each glycan group are given. Significant (uncorrected *p*-values <0.05) increases in relative peak area (in %) between risk drinkers and non-risk drinkers are marked with orange and in case of significant decrease are marked with blue. After sequential Bonferroni correction, only changes in GP9, GP31, GP33, and GP34 in men would be significant at an alpha level of 0.05. Structure abbreviations: all *N*-glycans have two core GlcNAcs; F at the start of the abbreviation indicates a core-fucose α1,6-linked to the inner GlcNAc; Mx, number (x) of mannose on core GlcNAcs; Ax, number of antenna (GlcNAc) on trimannosyl core; B, bisecting GlcNAc linked β1,4 to β1,3 mannose; Gx, number (x) of β1,4 linked galactose on antenna; Sx, number (x) of sialic acids linked to galactose.

The results of serum *N*-glycome (the relative proportion of the 46 GPs) in risk drinkers and non-risk drinkers are shown in Table 3. Among men, risk drinkers showed a lower abundance of GP5, GP8, GP9, GP10 and GP11, and a higher abundance of GP21, GP28, GP29, GP30, GP31, GP32, GP33, GP34 and GP42 than non-risk drinkers. A similar trend was observed in women, although only the lower abundance of GP8 and GP9 were statistically significant, as well as the higher abundance of GP28 and GP29. Furthermore, unlike men, a lower abundance of GP14 and a greater abundance of GP23 was observed among women risk drinkers (Table 3 and Figure 1).

Glycan features of serum *N*-glycans in risk drinkers and non-risk drinkers are shown in Table 4. Significant differences were only observed in men. The risk drinkers showed, compared to the non-risk drinkers, (a) a lower abundance of galactosylated (G0) and monogalactosylated (G1) glycans, together with a higher abundance of trigalactosylalated (G3) glycans; (b) a lower abundance of asialylated (S0) glycans together with a higher abundance of trisialylated (S3) glycans; (c) a lower abundance of diantennary (A2) glycans together with a higher abundance of triantennary (A3) glycans; and (d) a lower abundance of oligomannose (OM) glycans (Table 4).

Supplementary Table S3A–F shows the results of the serum *N*-glycome (individual GPs and aggregated in categories) and standard markers of risk drinkers (GGT, VCM, and CDT) in relation to the different levels of habitual alcohol consumption (0–9 g/week, 10–139 g/week, 140–279 g/week, and ≥280 g/week) in men and women. Among men, a significant trend towards a higher abundance of GP21, GP28, GP31, GP32, GP33, GP34, and trigalactosylated, trisialylated, triantennary, and outer fucose glycans in relation to increasing categories of alcohol consumption was confirmed (Supplementary Table S3A,B). The trend in women was less evident, although a significant trend towards a higher abundance of GP31 and GP32 in relation to increasing categories of alcohol consumption was observed, as well as GP3 and GP29 (Supplementary Table S3C,D).

**Figure 2:**
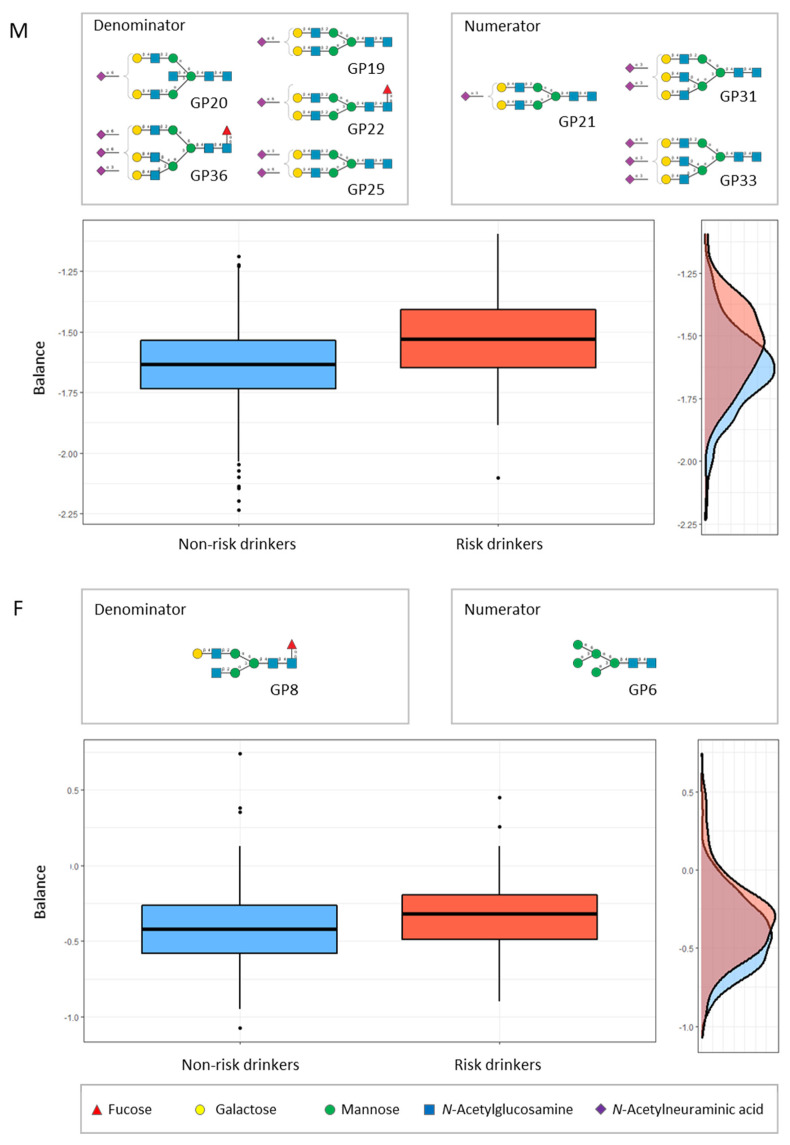
Description of the global *N*-glycan balance (or combination) for risk drinking using *selbal* algorithm [40]. The two groups of *N*-glycans that form the global balance (numerator and denominator) selected by *selbal* conditions are specified at the top of the plot. The box plot represents the distribution of the balance scores for risk drinkers and non-risk drinkers. The right part of the figure contains the density curve for each group. M (upper panel), men; F (lower panel), women.

**Figure 3:**
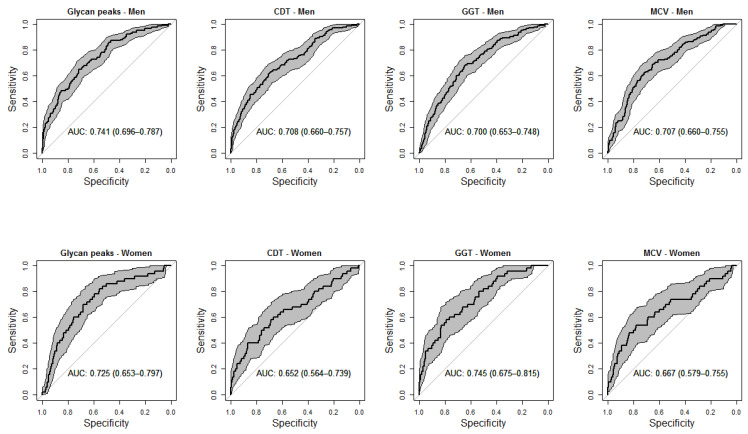
Receiver-operating characteristic (ROC) curves comparing the accuracy of serum *N*-glycan balance and commercial markers (serum carbohydrate-deficient transferrin [CDT], serum gamma-glutamyltransferase [GGT], and red blood cell mean corpuscular volume [MCV]) for the diagnosis of risk drinking in men (upper panels) and women (lower panels). All analyses were adjusted for age and smoking. Shadowed areas represent the 95% confidence interval of the ROC curve. Numbers in boxes represent the area under the curve with 95% confidence interval (within parentheses).

The *selbal* approach identified that a balance (or combination) consisting of GP21, GP31, and GP 33 (numerator) and GP19, GP20, GP22, GP25 and GP36 (denominator) was the best predictor combination for risk drinking in men, after adjusting for age and smoking (Figure 2). In the women, the best balance was GP6 (numerator), and GP8 (denominator) (Figure 2). Figure 3 compares the diagnostic accuracy (ROC curves) for risk drinking using the balance of GPs compared to the golden standard markers (GGT, MCV, and CDT), in all cases after adjusting for age and smoking in men and women. The AUC of the balance of GPs was at least the same as that of the GGT, MCV and CDT, without significant differences (Figure 3). In men, the combination of GPs with GGT increased the AUC of the latter from 0.70 to 0.75, increased the AUC of MCV from 0.70 to 0.76, and increased the AUC of CDT from 0.70 to 0.77. In women, the combination of GPs with GGT increased the AUC of the latter from 0.74 to 0.76, increased the AUC of MCV from 0.66 to 0.73, and increased the AUC of CDT from 0.65 to 0.73.

## 4. Discussion

The demonstration of the relationship between alcohol consumption and abnormal transferrin glycosylation dates back to 1979 and was the first commercial glycomic marker of alcohol abuse (CDT, carbohydrate-deficient transferrin) [17]. Since then, the diagnostic value of CDT has been extensively studied, but other potential glycomic markers of alcohol risk drinking have not been investigated until now. Recent technological development enabled systematic analysis of serum *N*-glycome composition in large epidemiological cohorts and clinical studies [8,26,27]. The present study shows that delineating the serum *N*-glycome can add diagnostic value to the usual markers of risk drinking and can form the basis on which future investigations of glycomic markers are planned.

The classic commercial markers of risk drinking (GGT, MCV, and CDT) showed differential performance in men compared to women. In general, these classic markers demonstrated high specificity but low sensitivity, as reported previously [2,3,4,5,6]. The sensitivity in our study was even lower compared to previous reports, possibly because the threshold for the definition of risk drinking was especially low, according to current guidelines. The sensitivity in men was highest for CDT and lowest for MCV. In women, the sensitivity was highest for GGT and lowest for the glycomic marker CDT. Something similar happened in the serum *N*-glycome study, in which the greatest differences were observed in men compared to women, for the distribution of the individual GPs and for the GPs grouped into categories by their common traits. The differences in the effects of alcohol between men and women are well known [42]. In addition, the proportion of risk drinkers is lower among women and high-risk drinking women have a lower amount of consumption than men. Moreover, the distribution of *N*-glycome is different in men and women [14]. For all these reasons, all analyses were stratified by sex.

In our study, male risk drinkers showed higher abundances of triantennary glycans (versus a lower abundance of biantennary glycans), a higher abundance of trigalactosylated glycans (versus a lower abundance of galactosylated and monogalactosylated glycans), than non-risk drinkers. Specifically, both triantennary (A3) trigalactosylated (G3) glycans such as GP31 (A3G3S[3,3]2) and GP33 (A3G3S[3,3,6]3) were significantly more abundant in risk drinkers compared to non-risk drinkers. Furthermore, the abundance of these GPs significantly increased with increasing alcohol intake among drinkers. Moreover, these two GPs formed part of the numerator of the best balance of GPs [40,41] that was associated with risk drinking. Among men, risk drinkers also showed a higher abundance of trisialylated (S3) glycans (versus a lower abundance of asialylated (G0) glycans). As transferrin is an abundant serum protein (the second most abundant serum glycoprotein after IgG) [26], it could be expected that the glycosylation-inhibiting effect of alcohol consumption on transferrin (i.e., CDT) will be reflected in the serum *N*-glycome. The beta-galactosidase alpha-2,6-sialyltransferase 1 (encoded by *ST6GAL1*) is a principal sialyltransferase responsible for the creation of the terminal α2,6-galactosyl linkages on galactose-containing substrates, i.e., the transfer of sialic acid residue to a terminal galactose residue. The *ST6GAL1* gene was found to be down-regulated by increasing alcohol intake and inhibited by acetaldehyde, the main by-product of ethanol metabolism; these are proposed to increase the low sialylated forms of transferrin (CDT) [22,23]. The human serum transferrin glycosylation mainly (96.5%) consists of the A2G2S2 structure [18]. *N*-glycans with an A2G2S[3,6]2 structure include GP24, GP25, and GP27. Of them, only GP25 showed a significant trend to a lower abundance with increasing alcohol consumption in men. Similar changes were not observed among women. Regarding individual GPs, the more consistent changes in men and women were a higher abundance of GP28 (FA2BG2S[6,6]2) and GP29 (A3G3S[3,6]2), and a lower abundance of GP8 (FA2[6]G1) and GP9 (FA2[3]G1) among risk drinkers. The possible meaning of these variations and the determination of which specific proteins (or other glycoconjugates) this glycosylation corresponds to should be addressed in future studies.

In addition to the pathophysiological interest (i.e., the specific alterations in the glycosylation process in relation to alcohol consumption), from a clinical point of view, it is interesting to obtain summary measures that may have practical applicability, in this case, diagnostic (detection of risk drinking). The usual methods for the determination of *N*-glycome display compositional data (i.e., proportions of some whole). The identification of global signatures that are predictive of the variable of interest (risk drinking) is an essential step toward the translation of serum *N*-glycome research to clinical practice. By means of *selbal*, a forward selection algorithm, we identified signatures consisting of two groups of *N*-glycans whose relative abundances, or balance, are predictive of the outcome [40]. Working with balances and, in general, with log-contrast functions preserves the scale-invariant principle for compositional data analysis [40]. For this purpose, we maintained sex stratification and further adjusted the results for age and smoking, two confounding variables that are associated with both risk drinking and *N*-glycome variations [15]. In men, we identified that the balance of the above-mentioned GP31, GP33, and GP20 (numerator) with GP19, GP20, GP22, GP25, and GP36 (denominator) showed an accuracy for the diagnosis of risk drinking which was at least as high as that of CDT, MCV and GGT. In women, the balance of GP6 (numerator) with the above-mentioned GP8 (denominator) also showed an accuracy for the diagnosis of risk drinking which was at least as high as that of the commercial markers CDT, MCV and GGT. Given that the combined use of markers is recommended for improving accuracy [4], we further observed that the addition of the GP balance to the classical markers improved their accuracy, both in men and women. It must be recognised, however, that the diagnostic accuracy of *N*-glycome, alone or in combination with GGT, MCV, and CDT still remains relatively low for the detection of risk drinking, as defined in the present study.

The study has strengths and limitations that should be acknowledged. The general population-based design may be considered a strength of the study. Investigation of analytes in the general population could help the interpretation of abnormal laboratory results in the disease, the definition of reference values, and the investigation of the influence of common factors, such as alcohol consumption in this case. Misclassification can occur when measuring alcohol consumption, which is a difficult discipline in itself. The system of standard drinking units is widely accepted, provides comparable results, and was validated in Spain [28]. To improve the ability to detect risk drinking, a combined definition was established with the AUDIT questionnaire, which was also validated in Spain [29,30,31]. Women show particular attitudes and effects with alcohol use disorders [43,44], for which specific thresholds were established in the definition of risk drinking for men and women. Temporal ambiguity in the possible cause–effect association is inherent to the cross-sectional design. Multivariate analyses were performed in order to adjust for confounding factors (age and smoking, in addition to sex). To the best of our knowledge, this is the first study that investigates the effects of alcohol on serum *N*-glycome.

## 5. Conclusions

In summary, in this article, we describe the variations in the *N*-glycome serum in relation to alcohol consumption. The determination of *N*-glycome has at least the same diagnostic value for risk drinking as conventional commercial markers and could provide additional information to them, although, as a whole, their diagnostic precision remains relatively low. Future studies will be necessary to determine whether the pattern of glycosylation of specific proteins can enhance the utility of glycomic clinical markers, beyond CDT.

## Figures and Tables

**Table 1:** General characteristics of risk drinkers and non-risk drinkers, stratified by sex.

Characteristic	Men			Women		
	Non-Risk Drinkers (n = 535)	Risk Drinkers (n = 143)	*p*-Value	Non-Risk Drinkers (n = 788)	Risk Drinkers (n = 50)	*p*-Value
Age (years)	53 (38–68)	48 (37–59)	0.002	53 (39–68)	50 (42–58)	0.226
Alcohol consumption (g/week)	60 (10–140)	320 (180–420)	<0.001	5 (0–30)	180 (100–220)	<0.001
Smokers (%)	107 (20.0)	65 (45.5)	<0.001	107 (13.6)	17 (34.0)	<0.001
Body mass index (kg/m^2^)	28.2 (25.2–31.3)	28.0 (25.3–31.3)	0.723	27.3 (23.8–31.3)	26.4 (23.3–31.5)	0.498

Data are presented as medians and interquartile ranges (between parentheses) or absolute numbers and percentages (between parentheses).

**Table 2:** Comparison of commercial markers between risk drinkers and non-risk drinkers, stratified by sex.

Marker	Men			Women		
	Non-Risk Drinkers (n = 535)	Risk Drinkers (n = 143)	*p*-Value	Non-Risk Drinkers (n = 788)	Risk Drinkers (n = 50)	*p*-Value
Serum GGT (IU/L)	26 (17–42)	40 (25–63)	<0.001	15 (11–23)	21 (15–54)	<0.001
Increased GGT n (%)	48 (9.0)	26 (18.2)	0.003	69 (8.8)	16 (32.0)	<0.001
RBC MCV (fL)	90 (87–93)	91 (88–95)	<0.001	89 (86–92)	91 (89–95)	0.001
Increased MCV n (%)	7 (1.3)	10 (7.1)	<0.001	1 (0.1)	5 (10.0)	<0.001
Serum CDT (%)	0.7 (0.6–0.9)	0.9 (0.7–1.7)	<0.001	0.6 (0.5–0.8)	0.8 (0.6–0.9)	0.004
Increased CDT n (%)	30 (5.6)	37 (25.9)	<0.001	4 (0.5)	4 (8.0)	<0.001

Data are medians and interquartile ranges (between parentheses) or absolute numbers and percentages (between parentheses). GGT, gamma-glutamyl transferase; RBC MCV, red blood cell mean corpuscular volume; CDT, carbohydrate-deficient transferrin. Increased GGT: values greater than 73 IU/L (men) or higher than 38 IU/L (women). Increased MCV: greater than 100 fL. Increased CDT: greater than 1.6%. The MCV was not available for 11 men and 5 women.

**Table 3:** Comparison of *N*-glycan peaks between risk drinkers and non-risk drinkers, stratified by sex.

Glycan Peak	Men			Women		
	Non-Risk Drinkers (n = 535)	Risk Drinkers (n = 143)	*p*-Value	Non-Risk Drinkers (n = 788)	Risk Drinkers (n = 50)	*p*-Value
GP1 (%)	0.12 (0.08–0.17)	0.10 (0.08–0.15)	0.096	0.11 (0.07–0.16)	0.10 (0.08–0.16)	0.936
GP2 (%)	0.04 (0.02–0.06)	0.04 (0.03–0.06)	0.078	0.03 (0.02–0.05)	0.03 (0.02–0.05)	0.721
GP3 (%)	0.08 (0.05–0.13)	0.07 (0.05–0.12)	0.782	0.07 (0.05–0.12)	0.07 (0.04–0.09)	0.520
GP4 (%)	0.06 (0.04–0.10)	0.05 (0.04–0.09)	0.236	0.06 (0.04–0.10)	0.06 (0.04–0.11)	0.871
GP5 (%)	2.41 (1.77–3.49)	2.12 (1.59–2.98)	0.010	2.25 (1.59–3.36)	2.04 (1.41–3.33)	0.317
GP6 (%)	1.04 (0.80–1.45)	0.96 (0.79–1.27)	0.077	1.03 (0.82–1.39)	1.00 (0.79–1.67)	0.732
GP7 (%)	0.09 (0.06–0.13)	0.09 (0.06–0.11)	0.409	0.09 (0.07–0.13)	0.09 (0.07–0.14)	0.835
GP8 (%)	1.95 (1.55–2.62)	1.72 (1.41–2.25)	0.003	1.92 (1.57–2.51)	1.60 (1.31–2.21)	0.016
GP9 (%)	1.09 (0.83–1.45)	0.93 (0.75–1.20)	<0.001	1.05 (0.82–1.35)	0.91 (0.75–1.12)	0.029
GP10 (%)	0.66 (0.52–0.90)	0.62 (0.48–0.78)	0.026	0.68 (0.55–0.86)	0.62 (0.52–0.99)	0.515
GP11 (%)	0.58 (0.48–0.72)	0.54 (0.43–0.65)	0.003	0.58 (0.48–0.70)	0.55 (0.47–0.73)	0.875
GP12 (%)	0.31 (0.23–0.40)	0.29 (0.23–0.38)	0.095	0.32 (0.24–0.42)	0.31 (0.23–0.40)	0.456
GP13 (%)	0.08 (0.05–0.10)	0.08 (0.05–0.10)	0.603	0.07 (0.06–0.10)	0.08 (0.05–0.10)	0.869
GP14 (%)	2.74 (2.25–3.33)	2.56 (2.12–3.16)	0.080	2.79 (2.25–3.48)	2.53 (1.96–3.31)	0.049
GP15 (%)	0.49 (0.40–0.61)	0.46 (0.39–0.56)	0.078	0.51 (0.40–0.63)	0.50 (0.41–0.67)	0.702
GP16 (%)	1.00 (0.87–1.15)	0.99 (0.89–1.15)	0.772	1.04 (0.90–1.20)	1.11 (0.97–1.19)	0.158
GP17 (%)	1.07 (0.88–1.23)	1.03 (0.89–1.20)	0.419	1.03 (0.87–1.19)	1.02 (0.82–1.20)	0.458
GP18 (%)	0.17 (0.13–0.21)	0.16 (0.13–0.20)	0.167	0.18 (0.13–0.22)	0.16 (0.12–0.21)	0.056
GP19 (%)	7.42 (6.87–8.11)	7.58 (6.90–8.16)	0.548	7.53 (6.90–8.15)	7.75 (7.04–8.16)	0.454
GP20 (%)	0.65 (0.57–0.61)	0.64 (0.58–0.70)	0.705	0.64 (0.57–0.71)	0.64 (0.57–0.72)	0.728
GP21 (%)	1.28 (1.11–1.48)	1.35 (1.17–1.54)	0.017	1.27 (1.10–1.43)	1.29 (1.10–1.47)	0.560
GP22 (%)	6.00 (5.26–6.83)	5.92 (5.21–6.78)	0.474	5.97 (5.23–7.17)	5.60 (4.98–6.92)	0.200
GP23 (%)	2.65 (2.16–3.29)	2.61 (2.24–3.40)	0.724	2.81 (2.33–3.52)	3.06 (2.60–3.92)	0.023
GP24 (%)	4.38 (3.92–4.92)	4.54 (4.03–4.96)	0.230	4.50 (4.02–4.99)	4.41 (3.99–5.03)	0.639
GP25 (%)	31.8 (29.2–33.8)	31.3 (29.0–33.7)	0.259	31.3 (29.2–33.2)	31.5 (28.2–32.9)	0.482
GP26 (%)	1.39 (1.22–1.59)	1.43 (1.27–1.63)	0.113	1.41 (1.23–1.59)	1.41 (1.22–1.58)	0.735
GP27 (%)	5.55 (4.77–6.36)	5.41 (4.63–6.21)	0.234	5.29 (4.62–5.97)	5.17 (4.53–5.58)	0.196
GP28 (%)	3.19 (2.66–3.81)	3.26 (2.87–4.16)	0.035	3.11 (2.65–3.64)	3.36 (2.77–4.03)	0.049
GP29 (%)	1.69 (1.42–1.94)	1.82 (1.53–2.06)	0.005	1.88 (1.62–2.09)	1.95 (1.69–2.31)	0.043
GP30 (%)	0.27 (0.22–0.34)	0.30 (0.25–0.35)	0.008	0.31 (0.25–0.36)	0.33 (0.24–0.37)	0.945
GP31 (%)	0.96 (0.83–1.14)	1.07 (0.89–1.25)	<0.001	1.10 (0.94–1.26)	1.14 (0.91–1.31)	0.674
GP32 (%)	0.65 (0.54–0.75)	0.70 (0.57–0.85)	0.007	0.58 (0.47–0.69)	0.59 (0.47–0.73)	0.590
GP33 (%)	0.88 (0.74–1.04)	0.97 (0.83–1.14)	<0.001	0.98 (0.84–1.14)	1.02 (0.90–1.20)	0.187
GP34 (%)	5.38 (4.34–6.52)	5.93 (4.90–7.17)	0.001	6.54 (5.44–7.59)	6.53 (5.67–7.42)	0.970
GP35 (%)	0.46 (0.37–0.55)	0.48 (0.38–0.58)	0.126	0.42 (0.34–0.51)	0.47 (0.36–0.56)	0.099
GP36 (%)	0.50 (0.40–0.61)	0.51 (0.44–0.61)	0.405	0.62 (0.50–0.78)	0.62 (0.48–0.77)	0.606
GP37 (%)	1.66 (1.39–2.04)	1.70 (1.41 (2.08)	0.384	1.81 (1.49–2.19)	1.77 (1.39–2.21)	0.491
GP38 (%)	3.76 (2.94–4.71)	3.95 (2.94–5.18)	0.260	2.70 (1.96–3.70)	3.23 (2.15–4.17)	0.070
GP39 (%)	0.44 (0.37–0.51)	0.45 (0.38–0.54)	0.309	0.45 (0.38–0.55)	0.46 (0.39–0.55)	0.797
GP40 (%)	0.44 (0.34–0.56)	0.43 (0.33–0.60)	0.494	0.38 (0.30–0.49)	0.43 (0.29–0.53)	0.273
GP41 (%)	0.44 (0.37–0.51)	0.46 (0.38–0.52)	0.131	0.45 (0.37–0.53)	0.44 (0.39–0.54)	0.751
GP42 (%)	0.25 (0.20–0.33)	0.28 (0.22–0.35)	0.016	0.29 (0.23–0.37)	0.29 (0.22–0.38)	0.984
GP43 (%)	0.39 (0.33–0.47)	0.41 (0.36–0.46)	0.166	0.43 (0.36–0.50)	0.42 (0.38–0.51)	0.939
GP44 (%)	0.22 (0.18–0.27)	0.23 (0.18–0.28)	0.262	0.21 (0.17–0.26)	0.22 (0.17–0.28)	0.939
GP45 (%)	0.27 (0.22–0.33)	0.27 (0.21–0.33)	0.890	0.22 (0.17–0.28)	0.23 (0.17–0.31)	0.506
GP46 (%)	0.17 (0.12–0.24)	0.17 (0.13–0.24)	0.526	0.15 (0.10–0.20)	0.17 (0.12–0.23)	0.155

Data are medians and interquartile ranges (between parentheses). GP, glycan peak. Uncorrected *p*-values are shown. Only *p*-values equal or lower than 0.001 would be significant at a 0.05 alpha level after correction by sequential Bonferroni adjustment.

**Table 4:** Comparison of *N*-glycan peaks, aggregated in groups, between risk drinkers and non-risk drinkers, stratified by sex.

Glycan Group	Men			Women		
	Non-Risk Drinkers (n = 535)	Risk Drinkers (n = 143)	*p*-Value	Non-Risk Drinkers (n = 788)	Risk Drinkers (n = 50)	*p*-Value
G0 (%)	3.32 (2.54–4.66)	2.95 (2.30–3.98)	0.014	3.15 (2.39–4.49)	2.89 (2.07–4.73)	0.337
G1 (%)	7.06 (6.02–8.53)	6.54 (5.82–7.75)	0.010	6.99 (6.15–8.18)	6.63 (5.65–7.81)	0.126
G2 (%)	68.3 (65.8–70.0)	67.6 (65.4–69.8)	0.185	67.7 (65.1–69.8)	67.5 (64.1–70.6)	0.951
G3 (%)	12.2 (10.5–14.3)	13.5 (11.9–15.2)	<0.001	14.1 (12.3–15.9)	14.2 (12.5–15.5)	0.736
G4 (%)	6.72 (5.68–7.96)	6.93 (5.61–8.74)	0.162	5.81 (4.71–6.93)	6.30 (4.96–7.61)	0.141
S0 (%)	12.0 (9.78–15.2)	10.7 (9.07–13.0)	0.002	11.8 (10.0–14.5)	10.4 (9.91–13.9)	0.069
S1 (%)	21.0 (19.1–22.5)	20.9 (19.4–22.5)	0.936	21.2 (19.6–23.0)	21.7 (19.7–23.2)	0.625
S2 (%)	49.7 (47.4–51.7)	50.0 (47.5–50.0)	0.865	49.2 (47.0–51.0)	49.7 (46.6–51.0)	0.723
S3 (%)	14.5 (12.8–16.2)	15.5 (13.8–17.2)	<0.001	14.9 (13.2–16.6)	14.8 (13.7–17.3)	0.484
S4 (%)	1.80 (1.51–2.13)	1.85 (1.61–2.19)	0.112	1.82 (1.48–2.12)	1.87 (1.59–2.22)	0.556
A1 (%)	1.08 (0.94–1.23)	1.10 (0.98–1.22)	0.159	1.06 (0.94–1.20)	1.04 (0.90–1.35)	0.913
A2 (%)	78.2 (76.1–80.0)	76.9 (75.3–79.2)	<0.001	77.5 (75.6–79.5)	77.0 (74.2–79.2)	0.196
A3 (%)	11.0 (9.51–12.8)	12.1 (10.5–13.5)	<0.001	12.7 (11.0–14.4)	12.7 (11.4–13.8)	0.986
A4 (%)	6.72 (5.68–7.96)	6.93 (5.61–8.74)	0.162	5.81 (4.71–6.94)	6.30 (4.96–7.61)	0.141
OM (%)	1.11 (0.92–1.44)	1.02 (0.85–1.24)	0.006	1.09 (0.92–1.39)	1.04 (0.88–1.54)	0.594
CF (%)	30.7 (27.4–34.3)	29.5 (26.4–33.2)	0.071	30.4 (27.2–33.7)	29.6 (26.8–33.2)	0.557
OF (%)	2.64 (2.26–3.06)	2.72 (2.33–3.16)	0.236	2.67 (2.28–3.13)	2.63 (2.15–3.12)	0.821

Data are medians and interquartile ranges (between parentheses). G0, agalactosylated; G1 monogalactosylated; G2, digalactosylated; G3, trigalactosylated; G4, tetragalactosylated; S0, asialylated; S1, monosialylated; S2, disialylated; S3, trisialylated; S4, tetrasialylated; A1, monoantennary; A2, biantennary; A3, triantennary; A4, tetraantennary; OM, oligomannose; CF, core-fucose; OF, outer-arm fucose. Uncorrected *p*-values are shown. Only *p*-values equal or lower than 0.003 would be significant at a 0.05 alpha level after correction by sequential Bonferroni adjustment.

## Data Availability

The data that support the findings of this study are available on reasonable request from the corresponding author. The data are not publicly available due to Spanish law restrictions.

## Supplementary Materials

The following are available online at https://www.mdpi.com/article/10.3390/biom12020241/s1. Table S1. The N-glycan composition of 46 peaks in control serum samples. Table S2. LC-MS Values detected for2-AB labelled N-glycans for normal human serum and two representative examples. Table S3. Comparison of individual *N*-glycan peaks, *N*-glycan peaks aggregated by features, and commercial markers of alcohol abuse among categories of alcohol consumption, in men and women.
