# Supplementary material for: Changes in Serum N-Glycome for Risk Drinkers: A Comparison with Standard Markers for Alcohol Abuse in Men and Women
Source: Biomolecules. 2022 Feb 1;12(2):241. doi: 10.3390/biom12020241 (PMC8961540; doi:10.3390/biom12020241)
Supplement: Supplementary file 1 [file biomolecules-12-00241-s001.zip › Table S1.pdf]

Table S1. The *N*-glycan composition of 46 peaks in control serum samples.

Peak	Name	Structure (major)	GU	Peak	Name	Structure (major)	GU
GP1	A1	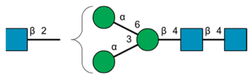	4.87	GP16	A2[3]BG1S[3]1	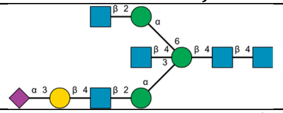	7.90
GP2	FA1	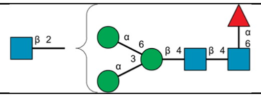	5.16	GP17	FA2[3]G1S[6]1	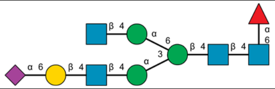	7.99
GP3	A1[6]G1	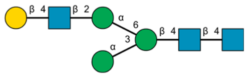	5.41	GP18	FA2[3]BG1S[3]1	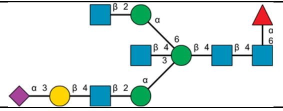	8.1
GP4	A2B	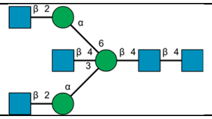	5.53	GP19	A2G2S[6]1	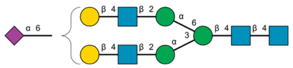	8.33
GP5	FA2	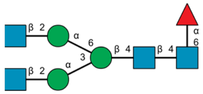	5.87	GP20	A2BG2S[6]1	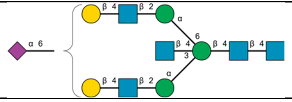	8.45
GP6	M5	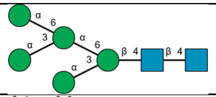	6.25	GP21	A2G2S[3]1	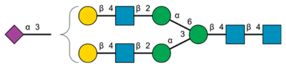	8.61
GP7	A2[6]BG1	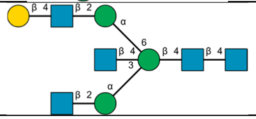	6.57	GP22	FA2G2S[6]1	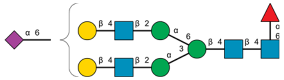	8.74
GP8	FA2[6]G1	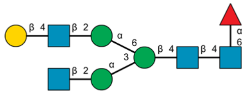	6.7	GP23	FA2BG2S[3]1	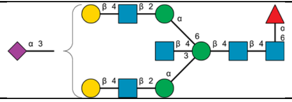	9.00
GP9	FA2[3]G1	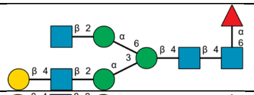	6.82	GP24	A2G2S[3,6]2	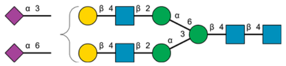	9.14
GP10	FA2[6]BG1	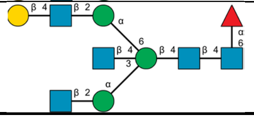	6.97	GP25	A2G2S[3,6]2	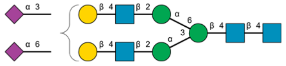	9.57

GP11	M6 D3	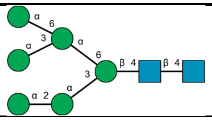	7.11	GP26	A2BG2S[3,6]2	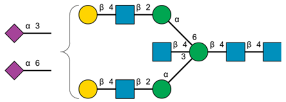	9.74
GP12	A1[3]G1S[3]1	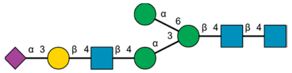	7.19	GP27	FA2G2S[3,6]2	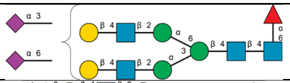	9.99
	A2G2	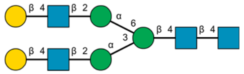		GP28	FA2BG2S[6,6]2	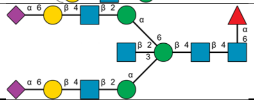	10.15
GP13	A2BG2	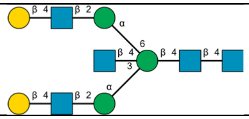	7.24	GP29	A3G3S[3,6]2	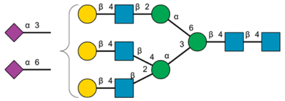	10.28
GP14	FA2G2	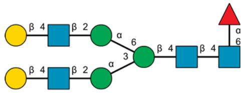	7.60	GP30	A4G4S[3]1	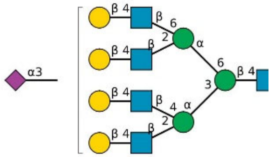	10.56
GP15	FA2BG2	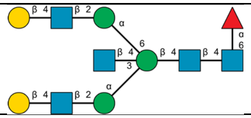	7.77	GP31	A3G3S[3,3]2	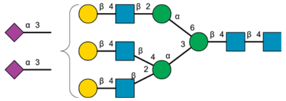	10.75
GP32	A3G3S[3,3,3]3	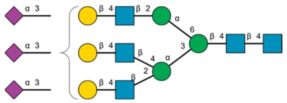	10.92	GP40	A4F1G3S[3,3,3]3	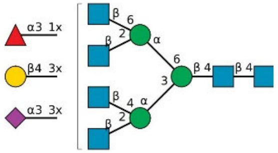	12.39
					A4F1G3S[3,3,6]3	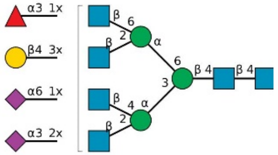	

					A4F1G3S[3,6,6]3	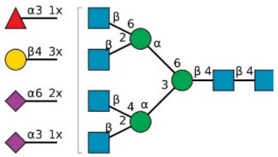	
GP33	A3G3S[3,3,6]3	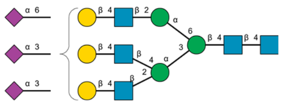	11.09	GP41	A4G4S[3,3,3,3]4	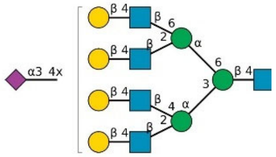	12.72
GP34	A3G3S[3,3,6]3	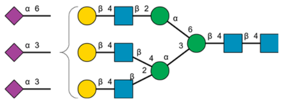	11.51	GP42	A4G4S[3,3,3,6]4	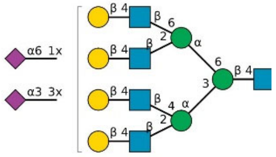	12.91
GP35	A3BG3S[3,3,3]3	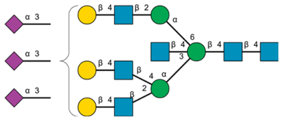	11.64	GP43	A4G4S[3,3,3,6]4	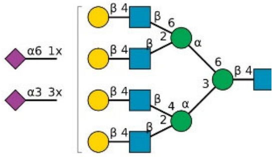	13.23
GP36	FA3G3S[3,6,6]3	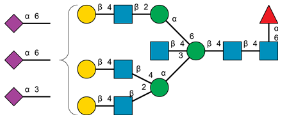	11.84	GP44	A4G4S[3,3,3,3]4	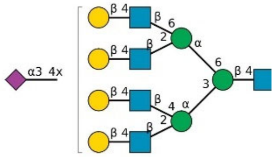	13.37

GP37	A3F1G3S[3,3,3]3	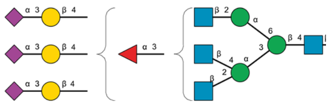	12.01	GP45	A4F1G4S[3,3,3,6]4	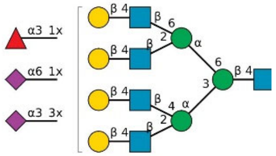	13.74
GP38	A4G4S[3,3,3]3	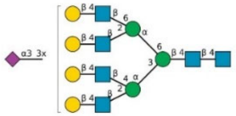	12.08	GP46	A4F3G4S[3,3,3,3]4	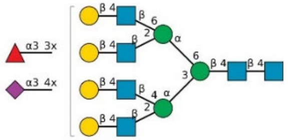	14.11
GP39	A4G4S[3,3,6]3<br>A4G4S[3,6,6]3	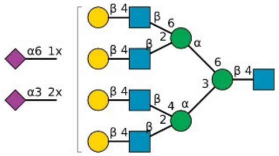<br>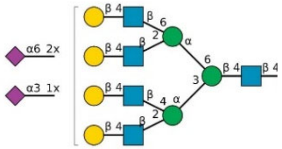	12.30				

Only main structures are shown in table and are defined based on assignments in Saldova et al. [12]. It is possible that on an individual basis other glycans are more prominant than those selected in this table.

Fucose Galactose Mannose *N*-Acetylglucosamine *N*-Acetylneuraminic acid
