# Supplementary material for: Changes in Serum N-Glycome for Risk Drinkers: A Comparison with Standard Markers for Alcohol Abuse in Men and Women
Source: Biomolecules. 2022 Feb 1;12(2):241. doi: 10.3390/biom12020241 (PMC8961540; doi:10.3390/biom12020241)
Supplement: Supplementary file 1 [file biomolecules-12-00241-s001.zip › Table S2.pdf]

Table S2. LC-MS Values detected for2-AB labelled *N*-glycans for normal human serum and two representative examples

Glycan Peak	GU	Glycan Structure	Monosaccharide composition	Monoisotopic mass	Theoretical mass (m/z)		Normal human serum control	Example 0001	Example 0002
						Ion	Experimental mass (m/z)	Experimental mass (m/z)	Experimental mass (m/z)
1	4.80	A1	HexNAc$_3$Hex$_3$	1233.4759	615.7307	[M-2H]$^{-2}$	-	615.7347	615.7307
2	5.09	M4	Hex$_4$	1192.4494	595.2174	[M-2H]$^{-2}$	-	-	595.2174
		FA1	dHex$_1$HexNAc$_3$Hex$_3$	1379.5338	688.7596	[M-2H]$^{-2}$	-	688.7495	688.7596
		A2	HexNAc$_4$Hex$_3$	1436.5553	717.2704	[M-2H]$^{-2}$	-	717.2668	717.2704
3	5.33	A1G1	HexNAc$_3$Hex$_4$	1395.5287	696.7571	[M-2H]$^{-2}$	-	-	696.7571
4	5.46	A2B	HexNAc$_5$Hex$_3$	1639.6346	818.8100	[M-2H]$^{-2}$	-	688.7495	818.8100
		A1G1	HexNAc$_3$Hex$_4$	1395.5287	696.7571	[M-2H]$^{-2}$	-	-	696.7571
5	5.80	FA2	dHex$_1$HexNAc$_4$Hex$_3$	1582.6132	790.2993	[M-2H]$^{-2}$	790.2950	790.2993	790.2950
6	6.17	M5	Hex$_5$	1354.5022	676.2438	[M-2H]$^{-2}$	676.2430	676.2537	676.2430
		FA2B	dHex$_1$HexNAc$_5$Hex$_3$	1785.6926	891.8390	[M-2H]$^{-2}$	891.8362	891.8362	891.8424
		FA1G1	dHex$_1$HexNAc$_3$Hex$_4$	1541.5866	769.7860	[M-2H]$^{-2}$	769.7758	769.7814	769.7928
		A2G1	HexNAc$_4$Hex$_4$	1598.6081	798.2968	[M-2H]$^{-2}$	798.2897	798.2995	798.3013
7	6.51	A2BG1	HexNAc$_5$Hex$_4$	1801.6875	899.8365	[M-2H]$^{-2}$	899.8354	899.8354	899.8478
8	6.64	FA2G1	dHex$_1$HexNAc$_4$Hex$_4$	1744.6660	871.3257	[M-2H]$^{-2}$	871.3254	871.3254	871.3132
		A2BG1	HexNAc$_5$Hex$_4$	1801.6875	899.8365	[M-2H]$^{-2}$	899.8354	899.8416	899.8478
		M4A1G1	HexNAc$_3$Hex$_5$	1557.5815	777.7835	[M-2H]$^{-2}$	777.7748	777.7806	777.7977
9	6.76	FA2G1	dHex$_1$HexNAc$_4$Hex$_4$	1744.6660	871.3257	[M-2H]$^{-2}$	871.3375	871.3132	871.3314
10	6.91	FA2BG1	dHex$_1$HexNAc$_5$Hex$_4$	1947.7454	972.8654	[M-2H]$^{-2}$	972.8658	972.8658	972.8530
11	7.05	FA2BG1	dHex$_1$HexNAc$_5$Hex$_4$	1947.7454	972.8654	[M-2H]$^{-2}$	972.8787	972.8658	972.8723
		M6	Hex$_6$	1516.5550	757.2702	[M-2H]$^{-2}$	757.2675	757.2787	757.2731
12	7.13	A1G1S1	HexNAc$_3$Neu5Ac$_1$Hex$_4$	1686.6241	842.3048	[M-2H]$^{-2}$	842.3096	842.3036	842.3036
		A2G2	HexNAc$_4$Hex$_5$	1760.6609	879.3232	[M-2H]$^{-2}$	879.3235	879.3356	879.3113
13	7.20	A1G1S1	HexNAc$_3$Neu5Ac$_1$Hex$_4$	1686.6241	842.3048	[M-2H]$^{-2}$	-	-	842.3036
		A2BG2	HexNAc$_5$Hex$_5$	1963.7403	980.8629	[M-2H]$^{-2}$	-	-	980.8536
14	7.53	M5A1G1	HexNAc$_3$Hex$_6$	1719.6344	858.8099	[M-2H]$^{-2}$	-	858.8076	858.7955
		FA2G2	dHex$_1$HexNAc$_4$Hex$_5$	1906.7188	952.3521	[M-2H]$^{-2}$	952.3431	952.3367	952.3367
		A2G1S1	HexNAc$_4$Neu5Ac$_2$Hex$_4$	1889.7036	943.8445	[M-2H]$^{-2}$	943.8297	943.8423	943.8171
		FA1G1S1	dHex$_1$HexNAc$_3$Neu5Ac$_2$ Hex$_4$	1832.6820	915.3337	[M-2H]$^{-2}$	-	915.3328	915.3328
15	7.70	FA2BG2	dHex$_1$HexNAc$_5$Hex$_5$	2109.7982	1053.8918	[M-2H]$^{-2}$	1053.8861	1053.8728	1053.8662
		M7	Hex$_7$	1678.6078	838.2966	[M-2H]$^{-2}$	-	838.3022	-
		A2BG1S1	HexNAc$_5$Neu5Ac$_1$Hex$_4$	2092.7829	1045.3842	[M-2H]$^{-2}$	-	-	1045.3936
16	7.85	A2BG1S1	HexNAc$_5$Neu5Ac$_1$Hex$_4$	2092.7829	1045.3842	[M-2H]$^{-2}$	1045.3737	1045.4003	1045.3737
		FA2G1S1	dHex$_1$HexNAc$_4$Neu5Ac$_1$ Hex$_4$	2035.7614	1016.8734	[M-2H]$^{-2}$	-	-	1016.8688
		M4A1G1S1	HexNAc$_3$Neu5Ac$_1$Hex$_5$	1848.6770	923.3312	[M-2H]$^{-2}$	923.3304	923.3367	923.3367
		M7	Hex$_7$	1678.6078	838.2966	[M-2H]$^{-2}$	838.3081	838.3081	838.3081
17	7.93	FA2G1S1	dHex$_1$HexNAc$_4$Neu5Ac$_1$ Hex$_4$	2035.7614	1016.8734	[M-2H]$^{-2}$	1016.8753	1016.8622	1016.8557
		FA2BG1S1	dHex$_1$HexNAc$_5$Neu5Ac$_1$ Hex$_4$	2238.8408	1118.4131	[M-2H]$^{-2}$	1118.4148	1118.4080	1118.4080

18	8.06	FA2BG1S1	dHex$_1$HexNAc$_5$Neu5Ac$_1$Hex$_4$	2238.8408	1118.4131	[M-2H]$^{-2}$	1118.3943	1118.4148	1118.4011
19	8.27	A2G2S1	HexNAc$_4$Neu5Ac$_1$Hex$_5$	2051.7563	1024.8709	[M-2H]$^{-2}$	1024.8574	1024.8771	1024.8640
		A3G3	HexNAc$_5$Hex$_6$	2125.7931	1061.8893	[M-2H]$^{-2}$	-	1061.8716	1061.8783
20	8.39	A2BG2S1	HexNAc$_5$Neu5Ac$_1$Hex$_5$	2254.8357	1126.4106	[M-2H]$^{-2}$	-	1126.4204	-
21	8.55	M5A1G1S1	HexNAc$_3$Neu5Ac$_1$Hex$_6$	2010.7298	1004.3576	[M-2H]$^{-2}$	-	-	-
		FA3G3	dHex$_1$HexNAc$_5$Hex$_6$	2271.8510	1134.9182	[M-2H]$^{-2}$	-	-	-
		M8	Hex$_8$	1840.6606	919.3230	[M-2H]$^{-2}$	-	-	-
		A2G2S1	HexNAc$_4$Neu5Ac$_1$Hex$_5$	2051.7563	1024.8709	[M-2H]$^{-2}$	-	-	-
22	8.67	FA2G2S1	dHex$_1$HexNAc$_4$Neu5Ac$_1$Hex$_5$	2197.8142	1097.8998	[M-2H]$^{-2}$	1097.8971	1097.9039	1097.8971
		M8	Hex$_8$	1840.6606	919.3230	[M-2H]$^{-2}$	-	919.3272	919.3334
23	8.92	FA2BG2S1	dHex$_1$HexNAc$_5$Neu5Ac$_1$Hex$_5$	2400.8936	1199.4395	[M-2H]$^{-2}$	1199.4496	1199.4211	1199.4282
24	9.08	A2F1G2S1	dHex$_1$HexNAc$_4$Neu5Ac$_1$Hex$_5$	2197.8142	1097.8998	[M-2H]$^{-2}$	1097.9106	-	1097.8835
		A2G2S2	HexNAc$_4$Neu5Ac$_2$Hex$_5$	2342.8518	1170.4186	[M-2H]$^{-2}$	1170.4243	1170.4314	1170.4103
		FA2G2S2	dHex$_1$HexNAc$_4$Neu5Ac$_2$Hex$_5$	2488.9097	1243.4476	[M-2H]$^{-2}$	1243.4333	1243.4333	1243.4261
25	9.50	A2G2S2	HexNAc$_4$Neu5Ac$_2$Hex$_5$	2342.8518	1170.4186	[M-2H]$^{-2}$	1170.4172	1170.4103	1170.4103
		M9	Hex$_9$	2002.7135	1000.3495	[M-2H]$^{-2}$	1000.3363	1000.3428	1000.3493
		A3G3S1	HexNAc$_5$Neu5Ac$_1$Hex$_6$	2416.8885	1207.4370	[M-2H]$^{-2}$	1207.4473	1207.4145	1207.4045
		A3BG3S1	HexNAc$_6$Neu5Ac$_1$Hex$_6$	2619.9679	1308.9767	[M-2H]$^{-2}$	-	-	1308.9713
26	9.67	FA3G3S1	dHex$_1$HexNAc$_5$Neu5Ac$_1$Hex$_6$	2562.9464	1280.4659	[M-2H]$^{-2}$	-	1280.4542	1280.4689
		FA3BG3S1	dHex$_1$HexNAc$_5$Neu5Ac$_1$Hex$_6$	2766.0258	1382.0056	[M-2H]$^{-2}$	-	-	-
		A2BG2S2	HexNAc$_5$Neu5Ac$_2$Hex$_5$	2545.9311	1271.9583	[M-2H]$^{-2}$	-	1271.9691	1271.9691
27	9.91	A3F1G3S1	dHex$_1$HexNAc$_5$Neu5Ac$_1$Hex$_6$	2562.9464	1280.4659	[M-2H]$^{-2}$	-	-	1280.4542
		FA2G2S2	dHex$_1$HexNAc$_4$Neu5Ac$_2$Hex$_5$	2488.9097	1243.4476	[M-2H]$^{-2}$	1243.4333	1243.4406	1243.4333
28	10.07	FA2BG2S2	dHex$_1$HexNAc$_5$Neu5Ac$_2$Hex$_5$	2691.9890	1344.9872	[M-2H]$^{-2}$	1344.9617	1344.9841	1344.9767
		A2F1G2S2	dHex$_1$HexNAc$_4$Neu5Ac$_2$Hex$_5$	2488.9097	1243.4476	[M-2H]$^{-2}$	1243.4623	1243.4406	1243.4551
		M9Glc	HexNAc$_1$Hex$_9$	2205.7928	1101.8891	[M-2H]$^{-2}$	-	-	-
29	10.19	A3G3S2	HexNAc$_5$Neu5Ac$_2$Hex$_6$	2707.9839	1352.9847	[M-2H]$^{-2}$	1352.9846	1352.9846	1352.9620
		A3BG3S2	HexNAc$_6$Neu5Ac$_2$Hex$_6$	2911.0633	1454.5244	[M-2H]$^{-2}$	-	-	-
30	10.47	A4G4S1	HexNAc$_6$Neu5Ac$_1$Hex$_7$	3655.3070	1826.6462	[M-2H]$^{-2}$	-	-	-
31	10.67	FA3G3S2	dHex$_1$HexNAc$_5$Neu5Ac$_2$Hex$_6$	2854.0419	1426.0137	[M-2H]$^{-2}$	-	1426.0101	1426.0333
		A3G3S2	HexNAc$_5$Neu5Ac$_2$Hex$_6$	2707.9839	1352.9847	[M-2H]$^{-2}$	1352.9998	1352.9696	1352.9620
32	10.83	A3G3S3	HexNAc$_5$Neu5Ac$_3$Hex$_6$	2999.0794	1498.5324	[M-2H]$^{-2}$	-	-	-
		A3F1G3S2	dHex$_1$HexNAc$_5$Neu5Ac$_2$Hex$_6$	2854.0419	1426.0137	[M-2H]$^{-2}$	-	-	1426.0178
		A4G4S1	HexNAc$_6$Neu5Ac$_1$Hex$_7$	3655.3070	1826.6462	[M-2H]$^{-2}$	-	-	-
33	11.02	A3G3S3	HexNAc$_5$Neu5Ac$_3$Hex$_6$	2999.0794	1498.5324	[M-2H]$^{-2}$	-	1498.5243	1498.5162
		A3BG3S3	HexNAc$_6$Neu5Ac$_3$Hex$_6$	3202.1587	1600.0721	[M-2H]$^{-2}$	-	-	-
34	11.43	FA3G3S3	dHex$_1$HexNAc$_5$Neu5Ac$_3$Hex$_6$	3145.1373	1571.5614	[M-2H]$^{-2}$	-	-	1571.5516

		FA3BG3S3	$dHex_1HexNAc_6Neu5Ac_3Hex_6$	3348.2166	1673.1010	$[M-2H]^{-2}$	-	-	-
		A4G4S2	$HexNAc_6Neu5Ac_2Hex_7$	3073.1161	1535.5508	$[M-2H]^{-2}$	-	-	-
		A3G3S3	$HexNAc_5Neu5Ac_3Hex_6$	2999.0794	1498.5324	$[M-2H]^{-2}$	1498.5560	1498.5243	1498.5322
35	11.57	A3BG3S3	$HexNAc_6Neu5Ac_3Hex_6$	3202.1587	1600.0721	$[M-2H]^{-2}$	-	-	-
36	11.77	FA3G3S3	$dHex_1HexNAc_5Neu5Ac_3Hex_6$	3145.1373	1571.5614	$[M-2H]^{-2}$	-	-	1571.5516
37	11.93	A3G3S3	$HexNAc_5Neu5Ac_3Hex_6$	2999.0794	1498.5324	$[M-2H]^{-2}$	1498.5560	1498.5162	1498.5243
		A3F1G3S3	$dHex_1HexNAc_5Neu5Ac_3Hex_6$	3145.1373	1571.5614	$[M-2H]^{-2}$	1571.5516	1571.5516	1571.5435
		FA3BG3S3	$dHex_1HexNAc_6Neu5Ac_3Hex_6$	3348.2166	1673.1010	$[M-2H]^{-2}$	-	-	-
38	12.00	A4G4S3	$HexNAc_6Neu5Ac_3Hex_7$	3364.2116	1681.0985	$[M-2H]^{-2}$	1681.1193	1681.0941	-
		A3F1G3S3	$dHex_1HexNAc_5Neu5Ac_3Hex_6$	3145.1373	1571.5614	$[M-2H]^{-2}$	1571.5435	1571.5516	1571.5110
39	12.21	A4G4S3	$HexNAc_6Neu5Ac_3Hex_7$	3364.2116	1681.0985	$[M-2H]^{-2}$	1681.1193	-	-
40	12.32	A4F1G3S3	$dHex_1HexNAc_6Neu5Ac_3Hex_6$	3348.2166	1673.1010	$[M-2H]^{-2}$	-	-	-
		A3F2G3S3	$dHex_2HexNAc_5Neu5Ac_3Hex_6$	3291.1952	1644.5903	$[M-2H]^{-2}$	-	1644.5774	-
41	23.57	A4G4S4	$HexNAc_6Neu5Ac_4Hex_7$	3655.3070	1826.6462	$[M-2H]^{-2}$	-	-	-
		A3F2G3S3	$dHex_2HexNAc_5Neu5Ac_3Hex_6$	3291.1952	1644.5903	$[M-2H]^{-2}$	-	-	-
		A4F2G3S3	$dHex_2HexNAc_6Neu5Ac_3Hex_6$	3494.2746	1746.1300	$[M-2H]^{-2}$	-	-	-
42	12.82	A4G4S4	$HexNAc_6Neu5Ac_4Hex_7$	3655.3070	1826.6462	$[M-2H]^{-2}$	-	-	-
43	13.14	A4G4S4	$HexNAc_6Neu5Ac_4Hex_7$	3655.3070	1826.6462	$[M-2H]^{-2}$	-	-	-
44	13.27	A4G4S4	$HexNAc_6Neu5Ac_4Hex_7$	3655.3070	1826.6462	$[M-2H]^{-2}$	-	-	-
		FA4G4S4	$dHex_1HexNAc_6Neu5Ac_4Hex_7$	3801.3649	1899.6752	$[M-2H]^{-2}$	-	-	-
		A4BG4S4	$HexNAc_7Neu5Ac_4Hex_7$	3858.3864	1928.1859	$[M-2H]^{-2}$	-	-	-
45	13.63	A4F1G4S4	$dHex_1HexNAc_6Neu5Ac_4Hex_7$	3801.3649	1899.6752	$[M-2H]^{-2}$	-	-	-
46	14.22	A4F2G4S4	$dHex_2HexNAc_6Neu5Ac_4Hex_7$	3947.4228	1972.7041	$[M-2H]^{-2}$	-	-	-
		A4F3G4S4	$dHex_3HexNAc_6Neu5Ac_4Hex_7$	4093.4807	2045.7331	$[M-2H]^{-2}$	-	-	-
		A4G4Lac1S4	$HexNAc_7Neu5Ac_4Hex_8$	4020.4392	2009.2123	$[M-2H]^{-2}$	-	-	-
