# Supplementary material for: Changes in Serum N-Glycome for Risk Drinkers: A Comparison with Standard Markers for Alcohol Abuse in Men and Women
Source: Biomolecules. 2022 Feb 1;12(2):241. doi: 10.3390/biom12020241 (PMC8961540; doi:10.3390/biom12020241)
Supplement: Supplementary file 1 [file biomolecules-12-00241-s001.zip › Table S3.pdf]

Table S3. Comparison of individual *N*-glycan peaks, *N*-glycan peaks aggregated by features, and commercial markers of alcohol abuse among categories of alcohol consumption, in men and women.

Table S3. (A) Comparison of N-glycan peaks among categories of alcohol consumption in men

	Alcohol consumption (g/week)				
**Glycan peak**	**0–9 (n = 123)**	**10–139 (n = 270)**	**140–279 (n = 163)**	**≥280 (n = 122)**	***p*–value**
GP1 (%)	0.11 (0.08–0.17)	0.11 (0.08–0.15)	0.12 (0.08–0.18)	0.11 (0.08–0.18)	0.807
GP2 (%)	0.03 (0.02–0.05)	0.04 (0.02–0.05)	0.04 (0.03–0.07)	0.04 (0.03–0.07)	<0.001
GP3 (%)	0.08 (0.05–0.14)	0.07 (0.05–0.11)	0.08 (0.05–0.16)	0.08 (0.05–0.16)	0.076
GP4 (%)	0.06 (0.04–0.10)	0.06 (0.04–0.10)	0.06 (0.04–0.10)	0.06 (0.04–0.09)	0.325
GP5 (%)	2.50 (1.97–3.62)	2.22 (1.63–3.13)	2.58 (1.77–3.73)	2.33 (1.74–3.28)	0.811
GP6 (%)	1.12 (0.88–1.46)	0.95 (0.76–1.28)	1.06 (0.81–1.66)	1.02 (0.83–1.41)	0.499
GP7 (%)	0.09 (0.06–1.13)	0.09 (0.06–0.12)	0.09 (0.07–0.13)	0.09 (0.07–0.12)	0.198
GP8 (%)	1.93 (1.48–2.57)	1.94 (1.57–2.49)	1.97 (1.52–2.77)	1.72 (1.42–2.28)	0.198
GP9 (%)	1.10 (0.82–1.45)	1.09 (0.84–1.42)	1.04 (0.81–1.39)	0.90 (0.75–1.31)	0.026
GP10 (%)	0.70 (0.52–0.92)	0.62 (0.50–0.81)	0.70 (0.53–0.96)	0.64 (0.52–0.88)	0.725
GP11 (%)	0.61 (0.49–0.75)	0.56 (0.47–0.68)	0.60 (0.49–0.75)	0.56 (0.43–0.68)	0.341
GP12 (%)	0.32 (0.25–0,43)	0.31 (0.24–0.40)	0.29 (0.21–0.37)	0.31 (0.23–0.39)	0.049
GP13 (%)	0.08 (0.06–0.10)	0.07 (0.05–0.10)	0.07 (0.05–0.10)	0.08 (0.06–0.10)	0.710
GP14 (%)	2.77 (2.21–3.36)	2.81 (2.31–3.41)	2.57 (2.16–3.26)	2.46 (2.10–3.03)	0.017
GP15 (%)	0.49 (0.40–0.62)	0.49 (0.39–0.62)	0.48 (0.38–0.58)	0.47 (0.40–0.58)	0.199
GP16 (%)	1.03 (0.90–1.19)	1.00 (0.87–1.13)	0.95 (0.87–1.14)	1.03 (0.91–1.19)	0.518
GP17 (%)	1.06 (0.88–1.21)	1.07 (0.89–1.20)	1.09 (0.86–1.25)	1.05 (0.87–1.23)	0.707
GP18 (%)	0.18 (0.12–0.23)	0.18 (0.14–0.22)	0.15 (0.12–0.20)	0.16 (0.12–0.20)	<0.001
GP19 (%)	7.59 (6.89–8.27)	7.46 (6.90–8.09)	7.39 (6.76–7.89)	7.54 (6.89–8.11)	0.501
GP20 (%)	0.64 (0.57–0.73)	0.66 (0.59–0.72)	0.62 (0.55–0.70)	0.65 (0.59–0.69)	0.122
GP21 (%)	1.27 (1.11–1.47)	1.30 (1.12–1.47	1.28 (1.11–1.54)	1.36 (1.17–1.52)	0.046
GP22 (%)	5.79 (5.16–6.81)	6.26 (5.46–7.02)	5.91 (5.16–6.67)	5.71 (5.08–6.57)	0.018
GP23 (%)	2.65 (2.13–3.40)	2.66 (2.24–3.24)	2.61 (2.18–3.37)	2.66 (2.18–3.26)	0.827
GP24 (%)	4.47 (3.96–5.01)	4.50 (4.07–4.98)	4.31 (3.80–4.78)	4.46 (3.96–4.92)	0.195
GP25 (%)	32.4 (29.8–34.1)	31.9 (29.6–33.9)	31.3 (28.9–33.5)	31.1 (29.0–33.5)	0.010
GP26 (%)	1.38 (1.23–1.62)	1.39 (1.22–1.62)	1.40 (1.21–1.55)	1.42 (1.27–1.61)	0.535
GP27 (%)	5.34 (4.59–6.12)	5.68 (4.87–6.47)	5.60 (4.80–6.40)	5,39 (4.61–6.10)	0.931
GP28 (%)	3.17 (2.67–3.78)	3.16 (2.62–3.77)	3.16 (2.67–3.92)	3.38 (2.92–4.18)	0.030
GP29 (%)	1.68 (1.41–2.00)	1.70 (1.46–1.93)	1.69 (1.44–2.02)	1.75 (1.49–2.06)	0.111
GP30 (%)	0.27 (0.21–0.33)	0.28 (0.22–0.34)	0.28 (0.21–0.34)	0.29 (0.25–0.36)	0.135
GP31 (%)	0.92 (0.82–1.14)	0.97 (0.82–1.12)	1.01 (0.84–1.17)	1.06 (0.91–1.19)	<0.001
GP32 (%)	0.64 (0.54–0.74)	0.63 (0.54–0.75)	0.68 (0.56–0.78)	0.70 (0.59–0.83)	0.002
GP33 (%)	0.88 (0.74–1.01)	0.89 (0.75–1.06)	0.89 (0.77–1.06)	0.97 (0.78–1.13)	0.022
GP34 (%)	5.37 (4.34–6.30)	5.38 (4.39–6.66)	5.49 (4.56–6.66)	5.90 (4.57–7.16)	0.045
GP35 (%)	0.46 (0.37–0.54)	0.46 (0.37–0.55)	0.47 (0.37–0.56)	0.48 (0.38–0.58)	0.286
GP36 (%)	0.47 (0.39–0.57)	0.51 (0.41–0.61)	0.51 (0.43–0.62)	0.51 (0.42–0.62)	0.104
GP37 (%)	1.62 (1.40–2.07)	1.66 (1.36–1.99)	1.72 (1.42–2.11)	1.75 (1.44–2.11)	0.102
GP38 (%)	3.59 (2.85–4.86)	3.72 (2.95–4.62)	3.96 (3.00–4.91)	3.94 (2.94–5.18)	0.124
GP39 (%)	0.42 (0.36–0.49)	0.43 (0.37–0.50)	0.47 (0.39–0.54)	0.46 (0.39–0.56)	<0.001
GP40 (%)	0.42 (0.33–0.51)	0.43 (0.33–0.54)	0.46 (0.35–0.59)	0.44 (0.34–0.60)	0.031
GP41 (%)	0.45 (0.37–0.54)	0.43 (0.37–0.50)	0.45 (0.37–0.51)	0.46 (0.38–0.53)	0.289
GP42 (%)	0.26 (0.20–0.33)	0.26 (0.20–0.33)	0.26 (0.21–0.33)	0.27 (0.21–0.34)	0.253
GP43 (%)	0.40 (0.33–0.46)	0.39 (0.33–0.45)	0.40 (0.33–0.48)	0.40 (0.35–0.46)	0.543
GP44 (%)	0.22 (0.18–0.26)	0.21 (0.18–0.27)	0.23 (0.17–0.28)	0.23 (0.17–0.28)	0.448
GP45 (%)	0.27 (0.21–0.34)	0.26 (0.22–0.33)	0.28 (0.22–0.34)	0.27 (0.21–0.33)	0.533
GP46 (%)	0.17 (0.12–0.22)	0.17 (0.12–0.24)	0.18 (0.13–0.25)	0.18 (0.13–0.23)	0.091

Data are medians and interquartile ranges (between parentheses). The orange colour represents higher abundance as alcohol consumption increases. The blue colour represents lower abundance as alcohol consumption increases. *p*-values were obtained with the Jonckheere-Terpstra test for trend. Uncorrected *p*-values are shown. Only *p*-values equal or lower than 0.001 would be significant at a 0.05 alpha level after correction by sequential Bonferroni adjustment.

Table S3. (B) Comparison of N-glycan peaks, aggregated in groups, among categories of alcohol consumption in men

	**Alcohol consumption (g/week)**				
**Glycan group**	**0–9 (n = 123)**	**10–139 (n = 270)**	**140–279 (n = 163)**	**≥280 (n = 122)**	***p*-value**
G0 (%)	3.44 (2.76–4.76)	3.04 (2.38–4.28)	3.47 (2.43–5.19)	3.23 (2.54–4.47)	0.818
G1 (%)	7.01 (6.06–8.66)	7.02 (6.01–8.18)	7.04 (6.02–8.67)	6.58 (5.82–7.88)	0.298
G2 (%)	68.4 (66.3–70.1)	68.9 (66.4–70.6)	67.6 (65.1–69.4)	67.4 (65.4–69.0)	<0.001
G3 (%)	12.2 (10.6–13.8)	12.3 (10.4–14.4)	12.7 (11.0–14.4)	13.5 (11.3–15.2)	0.005
G4 (%)	6.60 (5.62–7.90)	6.66 (5.76–7.89)	7.17 (5.57–8.43)	6.92 (5.62–8.55)	0.052
S0 (%)	12.3 (10.1–15.5)	11.5 (9.57–14.5)	11.8 (9.68–15.9)	10.9 (9.32–13.4)	0.223
S1 (%)	21.1 (19.0–22.6)	21.1 (19.6–22.7)	20.6 (19.0–22.1)	20.9 (19.4–22.5)	0.257
S2 (%)	49.4 (47.5–51.9)	50.0 (47.9–52.0)	49.4 (46.7–51.5)	49.8 (47.4–51.3)	0.231
S3 (%)	14.3 (12.7–16.0)	14.5 (12.8–16.1)	15.1 (13.1–16.5)	15.6 (13.8–17.1)	<0.001
S4 (%)	1.79 (1.49–2.08)	1.75 (1.53–2.06)	1.85 (1.56–2.18)	1.88 (1.48–2.20)	0.217
A1 (%)	1.08 (0.93–1.26)	1.06 (0.93–1.21)	1.11 (0.95–1.23)	1.12 (1.00–1.30)	0.008
A2 (%)	78.6 (76.4–80.2)	78.6 (76.1–80.1)	77.6 (75.8–79.4)	76.8 (75.2–79.1)	<0.001
A3 (%)	10.8 (9.50–12.4)	11.0 (9.46–13.1)	11.4 (9.91–13.0)	12.0 (10.1–13.5)	0.004
A4 (%)	6.60 (5.62–7.90)	6.66 (5.76–7.89)	7.17 (5.57–8.43)	6.92 (5.62–8.55)	0.052
OM (%)	1.18 (0.971.44)	1.04 (0.88–1.31)	1.15 (0.93–1.57)	1.07 (0.87–1.32)	0.790
CF (%)	30.4 (26.8–34–4)	30.4 (27.2–34.3)	30.8 (27.5–34.0)	29.5 (26.9–33.5)	0.750
OF (%)	2.58 (2.25–3.06)	2.57 (2.23–3.02)	2.75 (2.43–3.16)	2.78 (2.33–3.26)	0.011

Data are medians and interquartile ranges (between parentheses). G0, agalactosylated; G1 monogalactosylated; G2, digalactosylated; G3, trigalactosylated; G4, tetragalactosylated; S0, asialylated; S1, monosialylated; S2, disialylated; S3, trisialylated; S4, tetrasialylated; A1, monoantennary; A2, biantennary; A3, triantennary; A4, tetraantennary; OM, oligomannose; CF, core-fucose; OF, outer-arm fucose. The orange colour represents higher abundance as alcohol consumption increases. The blue colour represents lower abundance as alcohol consumption increases. *p*-values were obtained with the Jonckheere-Terpstra test for trend. Uncorrected *p*-values are shown. Only *p*-values equal or lower than 0.003 would be significant at a 0.05 alpha level after correction by sequential Bonferroni adjustment.

Table S3. (C) Comparison of N-glycan peaks among categories of alcohol consumption in women

	**Alcohol consumption (g/week)**				
**Glycan peak**	**0–9 (n = 423)**	**10–139 (n = 328)**	**140–279 (n = 78)**	**≥280 (n = 9)**	***p*-value**
GP1 (%)	0.11 (0.08–0.16)	0.11 (0.07–0.15)	0.13 (0.09–0.20)	0.12 (0.11–0.18)	0.214
GP2 (%)	0.03 (0.02–0.05)	0.03 (0.02–0.05)	0.04 (0.03–0.06)	0.04 (0.02–0.05)	0.218
GP3 (%)	0.07 (0.05–0.11)	0.08 (0.05–0.13)	0.08 (0.06–0.14)	0.11 (0.09–0.22)	0.036
GP4 (%)	0.06 (0.04–0.10)	0.06 (0.04–0.09)	0.07 (0.04–0.10)	0.06 (0.05–0.14)	0.563
GP5 (%)	2.21 (1.58–3.28)	2.18 (1.52–3.24)	2.69 (1.85–3.70)	2.85 (2.44–3.86)	0.280
GP6 (%)	1.02 (0.81–1.36)	1.01 (0.82–1.41)	1.12 (0.88–1.63)	1.54 (0.92–2.36)	0.199
GP7 (%)	0.09 (0.07–0.13)	0.09 (0.07–0.13)	0.09 (0.06–0.14)	0.10 (0.07–0.23)	0.541
GP8 (%)	1.92 (1.57–2.51)	1.90 (1.54–2.47)	1.81 (1.54–2.58)	1.84 (1.52–2.68)	0.341
GP9 (%)	1.06 (0.84–1.35)	1.02 (0.79–1.34)	1.03 (0.79–1.27)	1.09 (0.84–1.27)	0.160
GP10 (%)	0.68 (0.55–0.85)	0.67 (0.53–0.86)	0.67 (0.54–0.90)	0.82 (0.69–1.21)	0.909
GP11 (%)	0.58 (0.49–0.70)	0.57 (0.47–0.70)	0.59 (0.47–0.77)	0.68 (0.65–1.13)	0.787
GP12 (%)	0.31 (0.23–0.42)	0.33 (0.25–0.43)	0.30 (0.23.0.38)	0.36 (0.24–0.41)	0.871
GP13 (%)	0.07 (0.05–0.10)	0.08 (0.06–0.10)	0.08 (0.06–0.09)	0.10 (0.06–0.13)	0.682
GP14 (%)	2.80 (2.28–3.54)	2.84 (2.25–3.50)	2.39 (1.97–2.92)	2.79 (1.86–3.31)	0.011
GP15 (%)	0.51 (0.41–0.63)	0.51 (0.40–0.64)	0.46 (0.37–0.65)	0.51 (0.47–0.78)	0.292
GP16 (%)	1.02 (0.90–1.19)	1.05 (0.91–1.21)	1.06 (0.90–1.18)	1.13 (1.08–1.26)	0.142
GP17 (%)	1.04 (0.89–1.20)	1.01 (0.84–1.18)	1.04 (0.86–1.17)	1.13 (1.10–1.16)	0.278

GP18 (%)	0.18 (0.13–0.21)	0.18 (0.13–0.22)	0.17 (0.12–0.22)	0.18 (0.16–0.20)	0.746
GP19 (%)	7.51 (6.86–8.13)	7.62 (7.00–8.20)	7.48 (6.95–8.19)	7.76 (7.46–8.34)	0.169
GP20 (%)	0.64 (0.57–0.70)	0.64 (0.58–0.71)	0.63 (0.57–0.72)	0.58 (0.52–0.67)	0.850
GP21 (%)	1.26 (1.11–1.42)	1.26 (1.09–1.44)	1.30 (1.05–1.44)	1.43 (1.25–1.68)	0.656
GP22 (%)	6.04 (5.29–7.27)	6.00 (5.28–7.27)	5.47 (4.79–6.28)	5.15 (4.92–6.34)	0.005
GP23 (%)	2.84 (2.38–3.49)	2.85 (2.33–3.57)	2.78 (2.10–3.57)	3.65 (2.57–3.91)	0.545
GP24 (%)	4.43 (3.96–4.98)	4.59 (4.11–5.04)	4.30 (3.98–4.91)	3.88 (3.38–4.76)	0.293
GP25 (%)	31.2 (29.1–33.4)	31.4 (29.2–32.9)	31.1 (29.1–33.7)	28.2 (25.0–31.5)	0.929
GP26 (%)	1.42 (1.23–1.61)	1.40 (1.22–1.58)	1.39 (1.26–1.56)	1.49 (1.16–1.58)	0.326
GP27 (%)	5.35 (4.59–6.08)	5.23 (4.64–5.87)	5.28 (4.72–5.76)	4.52 (4.23–5.58)	0.178
GP28 (%)	3.07 (2.63–3.64)	3.16 (2.66–3.64)	3.18 (2.73–3.89)	3.47 (2.77–4.17)	0.141
GP29 (%)	1.83 (1.61–2.08)	1.91 (1.64–2.11)	1.94 (1.63–2.19)	1.83 (1.57–2.33)	0.029
GP30 (%)	0.30 (0.25–0.37)	0.31 (0.25–0.36)	0.32 (0.26–0.37)	0.30 (0.26–0.33)	0.799
GP31 (%)	1.08 (0.93–1.24)	1.13 (0.94–1.26)	1.20 (1.00–1.32)	1.02 (0.91–1.44)	0.004
GP32 (%)	0.57 (0.46–0.67)	0.59 (0.49–0.71)	0.61 (0.52–0.73)	0.57 (0.46–0.70)	0.037
GP33 (%)	0.97 (0.86–1.13)	0.99 (0.85–1.17)	0.99 (0.87–1.13)	0.91 (0.86–1.06)	0.150
GP34 (%)	6.60 (5.45–7.59)	6.49 (5.41–7.58)	6.55 (5.71–7.56)	6.69 (5.50–7.28)	0.718
GP35 (%)	0.41 (0.33–0.51)	0.43 (0.35–0.52)	0.42 (0.34–0.52)	0.37 (0.34–0.47)	0.228
GP36 (%)	0.63 (0.49–0.79)	0.61 (0.50–0.76)	0.65 (0.55–0.77)	0.60 (0.42–0.87)	0.956
GP37 (%)	1.81 (1.51–2.17)	1.78 (1.44–2.17)	1.90 (1.52–2.33)	1.92 (1.80–2.21)	0.605
GP38 (%)	2.67 (1.90–3.72)	2.83 (2.05–3.71)	2.83 (2.07–3.72)	1.77 (1.99–4.65)	0.164
GP39 (%)	0.44 (0.37–0.56)	0.45–0.38–0.53)	0.49 (0.42–0.58)	0.40 (0.38–0.53)	0.219
GP40 (%)	0.37 (0.29–0.49)	0.39 (0.30–0.49)	0.40 (0.30–0.51)	0.41 (0.32–0.59)	0.390
GP41 (%)	0.44 (0.37–0.53)	0.45 (0.38–0.53)	0.46 (0.39–0.56)	0.47 (0.44–0.53)	0.208
GP42 (%)	0.28 (0.23–0.37)	0.30 (0.23–0.37)	0.29 (0.24–0.34)	0.28 (0.22–0.40)	0.915
GP43 (%)	0.43 (0.36–0.51)	0.43 (0.36–0.50)	0.00 (0.38–0.52)	0.44 (0.41–0.46)	0.573
GP44 (%)	0.21 (0.16–0.26)	0.21 (0.17–0.26)	0.22 (0.17–0.26)	0.19 (0.16–0.22)	0.831
GP45 (%)	0.22 (0.17–0.28)	0.22 (0.17–0.28)	0.23 (0.18–0.28)	0.21 (0.14–0.34)	0.299
GP46 (%)	0.14 (0.10–0.20)	0.15 (0.10–0.20)	0.15 (0.11–0.21)	0.20 (0.12–0.22)	0.389

Data are medians and interquartile ranges (between parentheses). The orange colour represents higher abundance as alcohol consumption increases. The blue colour represents lower abundance as alcohol consumption increases. *p*-values were obtained with the Jonckheere-Terpstra test for trend. Uncorrected *p*-values are shown. Only *p*-values equal or lower than 0.001 would be significant at a 0.05 alpha level after correction by sequential Bonferroni adjustment.

Table S3. (D) Comparison of N-glycan peaks, aggregated in groups, among categories of alcohol consumption in women

	Alcohol consumption (g/week)				
**Glycan group**	**0–9 (n = 423)**	**10–139 (n = 328)**	**140–279 (n = 78)**	**≥280 (n = 9)**	***p*-value**
G0 (%)	3.10 (2.35–4.44)	3.08 (2.31–4.37)	3.72 (2.53–5.20)	4.17 (3.40–5.70)	0.242
G1 (%)	6.97 (6.18–8.07)	6.96 (6.01–8.21)	6.82 (5.94–8.24)	7.81 (6.97–8.10)	0.653
G2 (%)	67.9 (65.2–69.9)	67.8 (65.4–70.1)	67.2 (63.9–68.7)	66.8 (61.4–69.2)	0.075
G3 (%)	13.9 (12.2–15.7)	14.1 (12.4–15.9)	14.5 (12.7–16.2)	14.1 (12.1–15.0)	0.240
G4 (%)	5.69 (4.62–6.90)	5.89 (4.79–6.97)	6.06 (4.93–7.10)	5.45 (4.96–7.91)	0.175
S0 (%)	11.8 (10.1–14.5)	11.8 (9.83–14.3)	11.3 (9.72–15.19	13.5 (12.4–18.8)	0.649
S1 (%)	21.2 (19.6–23.0)	21.2 (19.6–23.0)	20.8 (18.5–22.7)	21.9 (19.8–22.2)	0.340
S2 (%)	49.3 (47.1–50.9)	49.2 (47.0–51.0)	49.7 (47.1–51.5)	47.3 (42.9–48.7)	0.787
S3 (%)	14.9 (13.0–16.4)	14.9 (13.3–16.7)	15.2 (13.4–17.3)	14.6 (13.7–14.8)	0.178
S4 (%)	1.79 (1.45–2.14)	1.83 (1.50–2.09)	1.82 (1.51–2.20)	1.92 (1.63–2.12)	0.499
A1 (%)	1.05 (0.94–1.19)	1.06 (0.94–1.21)	1.09 (0.97–1.25)	1.36 (1.12–1.43)	0.113
A2 (%)	77.7 (75.7–79.8)	77.6 (75.6–79.3)	76.9 (74.5–79.1)	75.6 (74.4–79.2)	0.030
A3 (%)	12.7 (10.8–14.3)	12.7 (11.1–14.3)	1.0 (11.6–14.5)	12.7 (11.2–13.3)	0.358
A4 (%)	5.69 (4.62–6.90)	5.89 (4.79–6.97)	6.06 (4.93–7.10)	5.45 (4.96–7.91)	0.175
OM (%)	1.09 (0.92–1.38)	1.08 (0.89–1.36)	1.14 (0.91–1.56)	1.44 (1.05–2.36)	0.684
CF (%)	30.5 (27.4–34.0)	30.2 (27.1–33.6)	29.3 (26.5–32.3)	32.7 (29.1–37.8)	0.099

OF (%)	2.66 (2.29–3.10)	2.64 (2.21–3.13)	2.79 (2.35–3.24)	3.04 (2.24–3.25)	0.327

Data are medians and interquartile ranges (between parentheses). G0, agalactosylated; G1 monogalactosylated; G2, digalactosylated; G3, trigalactosylated; G4, tetragalactosylated; S0, asialylated; S1, monosialylated; S2, disialylated; S3, trisialylated; S4, tetrasialylated; A1, monoantennary; A2, biantennary; A3, triantennary; A4, tetraantennary; OM, oligomannose; CF, core-fucose; OF, outer-arm fucose. The orange colour represents higher abundance as alcohol consumption increases. The blue colour represents lower abundance as alcohol consumption increases. *p*–values were obtained with the Jonckheere-Terpstra test for trend. Uncorrected *p*–values are shown. Only *p*–values equal or lower than 0.003 would be significant at a 0.05 alpha level after correction by sequential Bonferroni adjustment.

Table S3. (E) Comparison of commercial markers among categories of alcohol consumption in men

	Alcohol consumption (g/week)				
**Marker**	**0–9 (n = 123)**	**10–139 (n = 270)**	**140–279 (n = 163)**	**≥280 (n = 122)**	***p*–value**
Serum GGT (IU/L)	19 (14–33)	25 (17–39)	32 (23–47)	45 (29–68)	<0.001
Increased GGT n (%)	11 (8.9)	17 (6.3)	21 (12.9)	25 (20.5)	<0.001
RBC MCV (fL)	90 (86–92)	89 (86–92)	91 (87–94)	93 (89–96)	<0.001
Increased MCV n (%)	0 (0.0)	2 (0.8)	4 (2.5)	11 (9.2)	<0.001
Serum CDT (%)	0.6 (0.5–0.7)	0.7 (0.6–0.9)	0.8 (0.7–1.2)	1.0 (0.8–1.8)	<0.001
Increased CDT n (%)	2 (1.6)	10 (3.7)	19 (11.7)	36 (29.5)	<0.001

Data are medians and interquartile ranges (between parentheses). GGT, gamma–glutamyl transferase; RBC MCV, red blood cell mean corpuscular volume; CDT, carbohydrate-deficient transferrin. Increased GGT: values greater than 73 IU/L (reference value for men). Increased MCV: greater than 100 fL. Increased CDT: greater than 1.6%. *p*-values were obtained with the Jonckheere-Terpstra test for trend (for numerical variables) and with the chi-square test for trend (for categorical variables). The MCV was not available for 11 men.

Table S3. (F) Comparison of commercial markers among categories of alcohol consumption in women

	Alcohol consumption (g/week)				
**Marker**	**0–9 (n=423)**	**10–139 (n=328)**	**140–279 (n = 78)**	**≥280 (n = 9)**	***p*–value**
Serum GGT (IU/L)	15 (11–22)	15 (11–22)	24 (17–38)	67 (17–84)	<0.001
Increased GGT n (%)	34 (8.0)	25 (7.6)	20 (25.6)	6 (66.7)	<0.001
RBC MCV (fL)	88 (85–91)	90 (87–92)	91 (88–93)	90 (89–97)	<0.001
Increased MCV n (%)	1 (0.2)	1 (0.3)	2 (2.6)	2 (22.2)	<0.001
Serum CDT (%)	0.6 (0.5–0.7)	0.6 (0.5–0.8)	0.8 (0.6–0.9)	0.8 (0.7–1.0)	<0.001
Increased CDT n (%)	0 (0.0)	3 (0.9)	4 (5.1)	1 (11.1)	<0.001

Data are medians and interquartile ranges (between parentheses). GGT, gamma–glutamyl transferase; RBC MCV, red blood cell mean corpuscular volume; CDT, carbohydrate–deficient transferrin. Increased GGT: values greater than 38 IU/L (reference value for women). Increased MCV: greater than 100 fL. Increased CDT: greater than 1.6%. *p*-values were obtained with the Jonckheere-Terpstra test for trend (for numerical variables) and with the chi-square test for trend (for categorical variables). The MCV was not available for 5 women.
